# Aminobenzosuberone Scaffold as a Modular Chemical Tool for the Inhibition of Therapeutically Relevant M1 Aminopeptidases

**DOI:** 10.3390/molecules23102607

**Published:** 2018-10-11

**Authors:** Emmanuel Salomon, Marjorie Schmitt, Anil Kumar Marapaka, Athanasios Stamogiannos, Germain Revelant, Céline Schmitt, Sarah Alavi, Isabelle Florent, Anthony Addlagatta, Efstratios Stratikos, Céline Tarnus, Sébastien Albrecht

**Affiliations:** 1 Laboratoire d’Innovation Moléculaire et Applications, Université de Haute-Alsace, Université de Strasbourg, CNRS, 68093 Mulhouse, France; emmanuel.salomon@uha.fr (E.S.); marjorie.schmitt@uha.fr (M.S.); germain.revelant@hotmail.fr (G.R.); celine.schmitt85@gmail.com (C.S.); SALAVI@esta-groupe.fr (S.A.); celine.tarnus@uha.fr (C.T.); 2 Centre for Chemical Biology, CSIR-Indian Institute of Chemical Technology, Hyderabad 500007, Telangana, India; anilmarapaka@gmail.com (A.K.M.); anthonyaddlagatta@gmail.com (A.A.); 3 Academy of Scientific and Innovative Research (AcSIR), Rafi Marg, New Dehli 110001, India; 4 Protein Chemistry Laboratory, INRASTES, National Centre for Scientific Research Demokritos, Agia Paraskevi, 15310 Athens, Greece; a.stamogiannos@gmail.com (A.S.); stratikos@gmail.com (E.S.); 5 Molécules de Communication et Adaptation des Micro-organismes, Muséum National d’Histoire Naturelle, CNRS, 75231 Paris, France; isabelle.florent@mnhn.fr

**Keywords:** M1 aminopeptidases, selective inhibitors, aminobenzosuberone scaffold

## Abstract

The synthesis of racemic substituted 7-amino-5,7,8,9-tetrahydrobenzocyclohepten-6-one hydrochlorides was optimized to enhance reproducibility and increase the overall yield. In order to investigate their specificity, series of enzyme inhibition assays were carried out against a diversity of proteases, covering representative members of aspartic, cysteine, metallo and serine endopeptidases and including eight members of the monometallic M1 family of aminopeptidases as well as two members of the bimetallic M17 and M28 aminopeptidase families. This aminobenzosuberone scaffold indeed demonstrated selective inhibition of M1 aminopeptidases to the exclusion of other tested protease families; it was particularly potent against mammalian APN and its bacterial/parasitic orthologues *Ec*PepN and *Pf*AM1.

## 1. Introduction

Aminopeptidases (APs) constitute a group of exopeptidases with closely related activities, able to remove amino-acids from unblocked *N*-termini of peptide and protein substrates. Among them, metallo-aminopeptidases (metallo-APs) are widely distributed in organisms with representative members in animal cells, plant cells, fungi, parasites and bacteria [1,2]. Metallo-APs have been broadly sorted into two important subgroups on the basis of the number of zinc ions in their active site that are involved in catalysis as well as on their protein signatures and folding [1,2]. While some of these enzymes contain only one zinc, others possess a binuclear metal centre, usually referred as a co-catalytic unit [3,4]. Members of the M1 family belong to the subclan MA(E) of metallopeptidases [1], also called Glu-zincins and possess a single essential catalytic zinc ion and a common fold, related to their common ancestry that is related to thermolysin. Present mainly as monomers or homo-dimers (in mammals, mainly), metallo-APs occupy various cell compartments and although some are secreted, the vast majority operates as membrane bound ectoenzymes or cytosolic catalysts [2,5,6,7]. Their evolutionary history indicates that they are related to a complex and ancestral gene family that subsequently yielded series of more divergent sequences [8]. For this reason, their biochemical characterization and respective biological importance has, for a long time, been hindered by considerable confusion, due in part to their often large and sometimes overlapping substrate specificities as well as their very close biochemical properties. For the time being, we have a more precise picture of their highly specialized roles depending on their localization with many related paralogous members in mammals (12 in *H. sapiens*, 11 in rodents), whereas only a handful are known to be encoded in other species (3 or less; only one in *P. falciparum*) [1,9]. 

Human M1 paralogous members [1] have been named Leucyl-cystinyl aminopeptidase (*Hs*IRAP), endoplasmic reticulum aminopeptidase 1 (*Hs*ERAP1), endoplasmic reticulum aminopeptidase 2 (*Hs*ERAP2), aminopeptidase Q (*Hs*APQ), puromycin-sensitive aminopeptidase (*Hs*PSA), thyrotropin-releasing hormone-degrading ectoenzyme (*Hs*TRHDE), aminopeptidase A (*Hs*APA), aminopeptidase N (*Hs*APN), aminopeptidase O (*Hs*APO), leukotriene A_4_ hydrolase (*Hs*LTA_4_H), arginyl aminopeptidase B (*Hs*APB) and arginyl aminopeptidase B like 1 (*Hs*RNPL1) and their orthologues in *E. coli* (*Ec*PepN) and *P. falciparum* (*Pf*AM1). Their so far recognized biological implications are summarized in Table 1. Broadly, human M1 members play important biological roles in the renin-angiotensin system, the immune system as well as during the inflammatory process, while the bacterial and malarial members are involved in nutrient acquisition.

As a consequence from the definition of the M1 family, all members (the *MEROPS* database has so far identified 14718 amino acid sequences belonging to this M1 family) share critical characteristics [18,19]. Sequences alignment of a selection of M1 aminopeptidases highlights the conserved active site residues for substrate recognition and peptide bond hydrolysis that is, the zinc binding motif **H**Exx**H**x_18_**E**, the substrate binding motif, including the **GxMEN** domain and an extra **E** or **Q** distal residue, responsible for the recognition and binding of the key free amino terminus of peptide substrates, as well as the catalytic **E** residue (part of H**E**xxH motif) and a distal **Y** residue essential for transition state stabilization in the closed conformations of M1 aminopeptidases (Figure S1) [1]. 

Although overall amino acid sequence similarity may fall down to a mean value of 20%, a highly conserved 3D structure, reminding that of a sea-horse with 4 domains, has been found for all these M1 APs. Domain I folds as a twisted β-barrel of 200 aa, domain II corresponds to the ancestral thermolysin-like fold, domain III is a beta sandwich built with 2 β-sheets (absent in *Hs*LTA_4_H) and domain IV is a prominent bowl-like structure composed of α-helices (330 aa) which contains the active site channel entrance (about 40 Å long and 12 Å large) [20]. 

Dynamic conformational ensembles, involving inter-domains movements, are crucial for catalysis and various conformational states are particularly marked for mammalian enzymes. They include active site opening and closure, particularly important for *Hs*ERAP1, *Hs*IRAP and mammalian APN [20,21,22,23,24], suggesting that the peptide-binding channel could accommodate variously exposed *N*-termini of peptides and/or proteins. The influences of protein dimerization on active site accessibility and specificity as well as on various moonlighting functions have still to be more extensively studied for all M1 family of APs [23,25,26]. 

Substrate specificities have also been responsible for the confusion in biological roles attributed to these enzymes; as illustrated in Table 2, these substrate specificities are rarely discriminant with overlapping specificities being the rule and specific specificities the rare exceptions.

**Figure 1:**
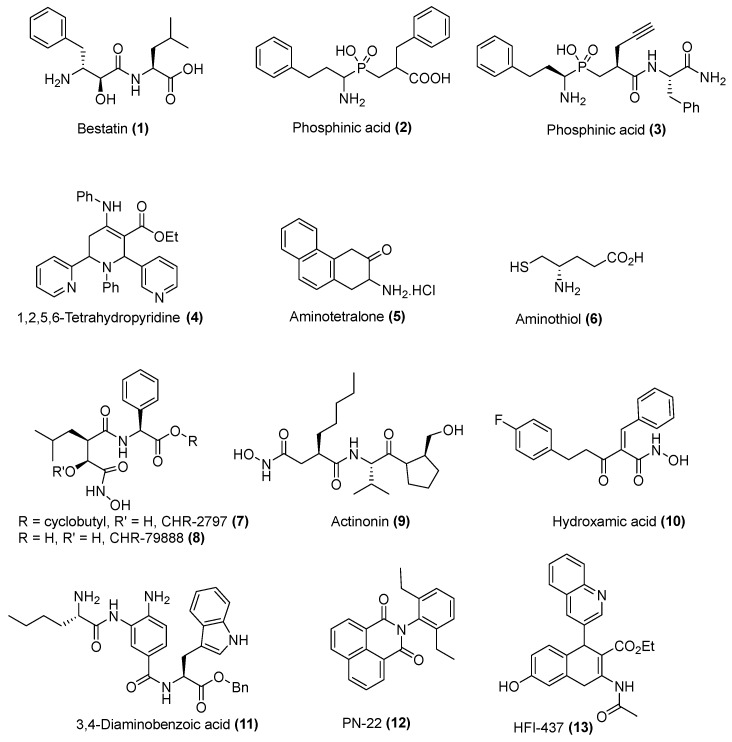
Representative inhibitors of M1 aminopeptidases (non-exhaustive list) For more detailed structures see references [11,28,29,30,31].

This common substrate specificity, together with a very conserved structure and catalytic mechanism, makes the development of highly selective inhibitors of a given metallo-aminopeptidase obviously challenging [11,28,29,30,31]. Thereby the most notable inhibitors, depicted in Figure 1, consist in tetrahedral intermediate mimics, as for the widely used bestatin [32] and phosphinic [33]/phosphonic [34] acid derivatives, or zinc-chelating group inserted in a peptide-like scaffold, as for hydroxamic acids [35,36,37]. 

In Table 3, we report inhibition values for several members of the M1 aminopeptidase family and for three selected bi-metallic aminopeptidases, since selectivity towards these other metallo-aminopeptidases remains of major concern and is indeed currently poorly documented. 

Inhibitors targeting mammalian APN have been the most extensively developed, since mammalian APN was one of the first metallo-aminopeptidase to be biochemically and enzymatically characterized (although many of its first assigned biological functions were later demonstrated to involve other related M1 paralogous enzymes). Among them, Bestatin **1** has been very often misused as a “specific” inhibitor of this peculiar APN enzyme, since it turns out to be a highly potent inhibitor of bimetallic enzyme families and a quite broad “unspecific” inhibitor of monometallic M1 aminopeptidases. But overall, it appears obvious that the development of M1 aminopeptidase inhibitors remains mainly in its infancy.

Our research group has previously reported the development of potent and selective inhibitors of APN, a human M1 aminopeptidase involved in angiogenesis and tumour metastasis [57]. This work allowed us to identify aminobenzosuberone moiety as a promising scaffold that demonstrates an exceptional selectivity towards mono-metallic aminopeptidases [42,58,59,60,61]. In the present work, we have closely considered the evaluation of these inhibitors on a number of potential interesting targets within the M1 family of aminopeptidases and established their inhibition profiles. We have reported herein an improvement of the aminobenzosuberone synthetic route and the biological evaluation of this family of non-peptidic compounds against 6 human and 2 bacterial/parasitic M1 aminopeptidases, plus members of the M17 and M28 families. We have also verified the observed selectivity on a panel of proteinases, which includes representative members of aspartic, cysteine, metallo and serine endopeptidases. 

## 2. Results

### 2.1. Chemistry

**Scheme 1:**
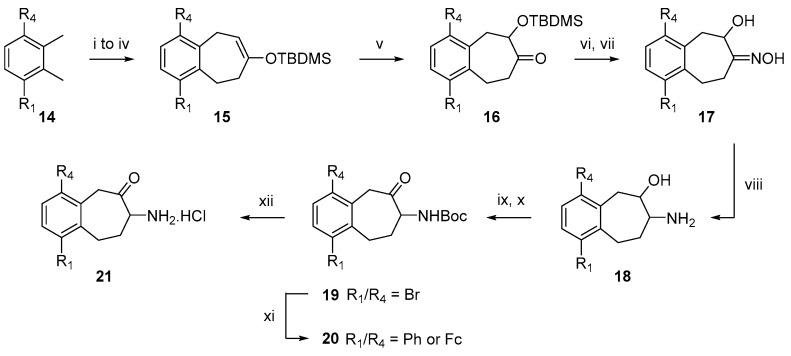
Synthetic scheme of racemic substituted 7-amino-5,7,8,9-tetrahydrobenzocyclohepten-6-one hydrochloride salts. Reagents and conditions: (i) NBS, AIBN, CCl_4_, reflux, 3 h. (ii) Dimethyl-1,3-acetonedicarboxylate, NBu_4_Br, NaHCO_3_ 1M aq., CH_2_Cl_2_, 40 °C, overnight. (iii) H_2_SO_4_, CH_3_CN, reflux, overnight. (iv) TBDMSOTf, NEt_3_, CH_2_Cl_2_, 25 °C, 2 h. (v) Oxone^®^, NaHCO_3_, acetone, CH_2_Cl_2_, 25 °C, 3 h. (vi) NH_2_OH. HCl, pyridine, 25 °C, 5 h. (vii) TBAF, THF, 25 °C, 2 h. Potential regioisomers may be separated at this point. (viii) NaBH_4_, CoCl_2_·6H_2_O, MeOH, −30 °C to 25 °C, 2 h. (ix) Boc_2_O, Na_2_CO_3_, MeOH, 25 °C, overnight. (x) Dess-Martin Periodinane, CH_2_Cl_2_, 25 °C, 2 h. (xi) PhB(OH)_2_ or FcB(OH)_2_, Pd(PPh_3_)_4_, K_2_CO_3_, DME, H_2_O, MW 125 °C, 30 min. (xii) 2M HCl in Et_2_O, 25 °C, overnight or for **21d**: TFA, CH_2_Cl_2_, 0 °C to RT, 20 min.

The aminobenzosuberone’s synthetic route, based on our previously described procedure [42,58,60], was optimized to improve reproducibility and avoid the appearance of side-products (Scheme 1). Indeed, silyl enol ether **15**, obtained in four steps from commercially available product **14**, was submitted to a Rubottom oxidation to yield the key intermediate silyloxy-ketone **16**. This reaction was classically performed by action of *m-*CPBA but regular and unpredictable failure with this reagent led us to consider alternative oxidizing agents. The best results were obtained with in situ generated dimethyl dioxirane (DMDO) [62,63], which gave only the expected product **16** in a nearly quantitative and reproducible yield (up to 95%). The second optimized step was the conversion of the hydroxy-oxime **17** to its corresponding amino-alcohol **18**. Our previously described method for this reduction consisted of a hydrogenation over Raney nickel [42,58]. Although the procedure worked efficiently, it was very dependent on the quality and supply of Raney nickel. Moreover, in the case of brominated compounds it required a careful monitoring to avoid any aromatic debromination. Thus, several conditions were investigated to obtain compound **18** as Mg/ammonium formate [64], SmI_2_ [65], BH_3_·THF [66], LiAlH_4_ [67] or NaBH_4_/NiCl_2_·6H_2_O [68]. Promising results were obtained with this latter but a small amount of debrominated product was still observed. Therefore, addition of NiCl_2_·6H_2_O was successfully replaced by addition of CoCl_2_·6H_2_O and the expected amino compound was solely obtained with no effect on aromatic bromine, even without strict monitoring of the reaction progress and in acceptable yields (60–80%).

### 2.2. In Vitro Inhibition of A Representative Panel of M1 Aminopeptidases

We have confirmed and extended the outstanding selectivity of aminobenzosuberone derivatives for M1 aminopeptidases over most other classes of proteinases (aspartic, cysteine, metallo and serine endopeptidase) with IC_50_ value >> 30 µM in all testes cases (Table S1). As expected, the bimetallic aminopeptidases from the M17 (*Pf*AM17) and M28 (A*AP*) families were not or only very weakly inhibited by the tested compounds (Table 4).

Among M1 aminopeptidases, the most potent inhibitory values remained on mammalian APN, with a subnanomolar K_i_ value for compound **21i** (K_i_ = 60 pM). Note that *Hs*LTA_4_H was not inhibited by this low-molecular weight scaffold, except for **21c** (K_i_ = 19 µM). The SAR determined for mammalian APN was very similar for its M1 orthologues from microorganisms, *Ec*PepN and *P. falciparum Pf*AM1, with an improved affinity when the aminobenzosuberone scaffold was substituted in C-1 or C-4 position with a hydrophobic functional group (for instance **21i** exhibited K_i_ values of 50 nM and 30 nM against *Ec*PepN and *Pf*AM1, respectively). The influence of bromine in C-1 position remained similar for the enzymes of both microorganisms with a 10 fold increase in potency when compared to the unsubstituted derivative **21a**. The main differences relied on the lack of synergistic effect (100-fold more potent for APN) when both positions were occupied by these functions (Ph and/or Br). Compounds **21c** and **21i** showed similar potencies on *Pf*AM1 (K_i_ values of 50 nM and 30 nM, respectively) and **21i** was only 3 times more potent than **21c** on *Ec*PepN.

As regard to human enzymes, *Hs*PSA is well-inhibited with K_i_ values of 55 and 21 nM for **21c** and **21i**, respectively. Within the oxytocinase subfamily, compounds were less active with inhibitory potencies in the micromolar range, except for **21h** on *Hs*IRAP (K_i_ = 34 nM). Generally, the inhibitors were less potent on *Hs*ERAP2 and *Hs*ERAP1 even if a similar trend emerged in the SAR studies. Actually, substitution at C-4 position seemed to be more efficient than substitution in the C-1 position, **21c** was usually more potent than **21f** and 1,4-disubstituted derivatives **21h** and **21i** displayed the most interesting binding affinity with a precise substitution pattern to achieve an excellent potency. 

## 3. Discussion

### 3.1. Structure-Based Analysis of the Structure-Activity Relationships

In the present work, we focused on subtle amino acid sequence differences in the active site that may explain the various inhibition values experimentally determined for the selected M1 aminopeptidases. In addition, conformational dynamics have been considered for some members of this family among the structures captured during various crystallization processes [20,21,24,70]. Data remain scarce due to the small number of solved 3D structures and no general statements could be outlined, nevertheless our SAR studies enabled to precise the structural environment required for potent inhibition by the aminobenzosuberone derivatives of a given enzyme within the M1 family. Our discussion will essentially focus on “closed” conformation of different aminopeptidases.

The determined structures from various species indicate that the active site of a M1 aminopeptidase is buried inside the protein and its zinc binding site may be located in a spacious cavity with wide openings, or located in a small and gated compartment as it is the case for bacterial/parasite orthologues [71]: this was in agreement with the active site’s cavity volume of different M1 aminopeptidases analysed by KVFinder (Table S2 and Figure S2) [72]. Local or intradomain/interdomain movements can cause enlargement or contraction of the active site as encountered for *Ec*PepN or mammalian APN [20,25,71,73].

**Figure 2:**
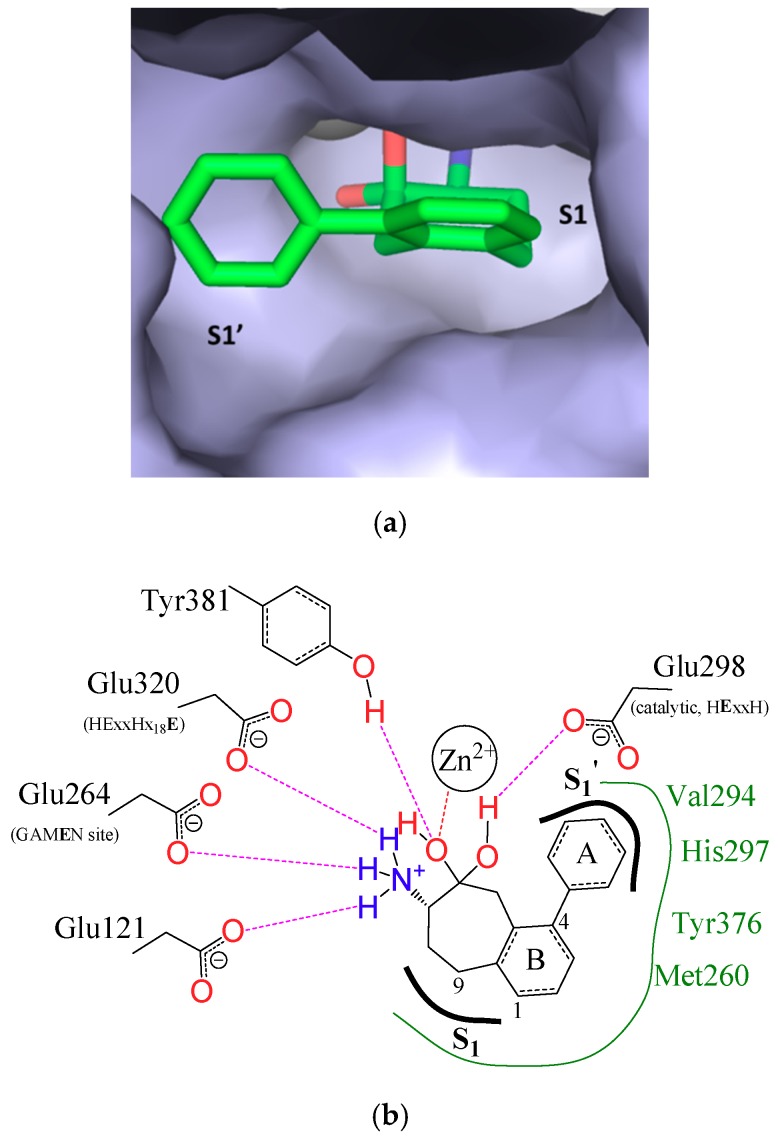
Interactions of aminobenzosuberone derivative **21c** with *Ec*PepN. (**a**) Surface representation of compound **21c** in the active site of *Ec*PepN (PDB 5MFS). The binding pocket is shown in light-blue surface representation and the zinc ion is represented by a grey sphere (prepared using PyMOL Molecular Graphics System). (**b**) Schematic representation of compound **21c** binding mode in the active site of *Ec*PepN. Hydrogen bond and metal interactions are shown with dotted purple and red lines, respectively. Green lines represent the hydrophobic residues located in the active site [74].

However, the architecture and amino acid composition of the active site are highly conserved within the whole family [1], which permits to predict an identical binding mode of the aminobenzosuberone ligand in the various M1 aminopeptidases similar to the previous model reported for mammalian APN [60] or *Ec*PepN-aminobenzosuberone co-crystallized structures (Figure 2) [74].

### 3.2. S1 Subsite

The S1 subsite is always described as a cylinder formed by hydrophobic residues and capped by two polar amino acids [75,76]. In Table 5, key residues of this constitutive recognition site are summarized. Interestingly, a highly variable residue (highlighted in “red”), both in steric size and polarity is located at the entrance of this pocket and may contribute to the diversity of S1 specificities (Table S3 and Figure S3) [75].

**Figure 3:**
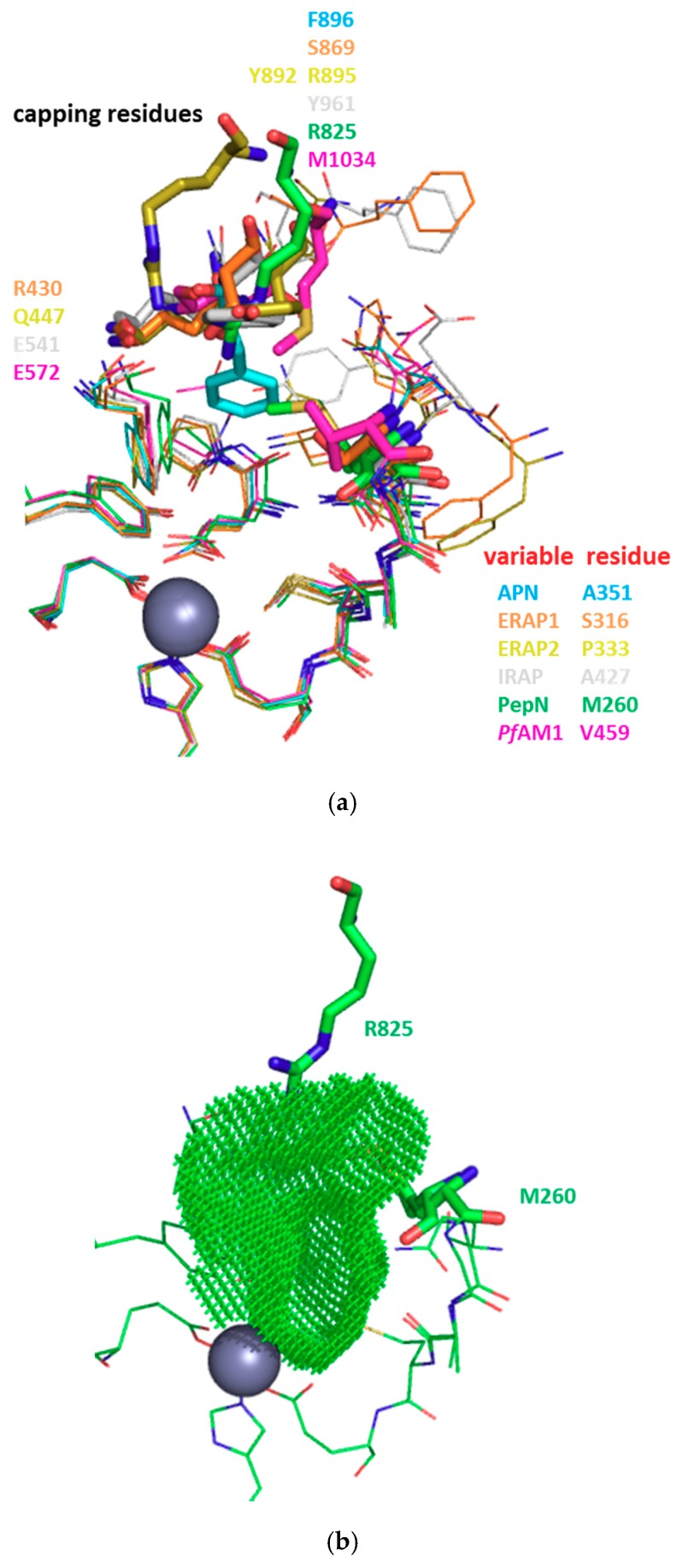
Diversity in the S1 subsite of different M1 aminopeptidases (**a**) Superimposition of the protein backbone of S1 subsite of different M1 APs: *Hs*APN (cyan, PDB 4FYT), *Hs*ERAP1 (orange, PDB 2YD0), *Hs*ERAP2 (yellow, PDB 4JBS), *Hs*IRAP (white, PDB 5MJ6), *Ec*PepN (green, PDB 5MFS), *Pf*AM1 (magenta, PDB 3EBH). (**b**) Surface representation of the S1 subsite of *Ec*PepN (PDB 5MFS). The catalytic zinc ion is shown as grey sphere. Residues involved in S1 plasticity are shown in stick. Images generated with KVFinder plugin in PyMOL.

The aforementioned variable residue may be flexible upon ligand binding or might be sterically hindered, restricting thus the S1 subsite entrance (Figure 3 and for more details, Figure S3). For instance, in *Ec*PepN it corresponds to Met260 (PDB 2DQM or 5MFS) with a temperature factor value of 28–37 Å^2^, in agreement with a local significant flexibility of its side chain, which acts as a cushion to accommodate various side chains in P1 position of a substrate/ligand. This local movement of Met residue can cause the contraction of the active site cavity. KVFinder analysis revealed that the cavity volume of *Ec*PepN-bestatin 1 complex (PDB 2DQM) was higher (638.5 Å^3^), compared to 531.25 Å^3^ for the *Ec*PepN-aminobenzuberone **21c** complex (PDB 5MFS) (Table S2). In this latter complex, this Met260 residue was found at two closed positions and moved slightly inward the S1 subsite, interrupting and shortening its typical cylindrical shape. The distance between the sulphur atom of Met260 and the centroid of the aromatic ring of the aminobenzosuberone scaffold is 5.3–5.7Å, which is consistent with sulphur-carbon van der Waals contacts and the sulphur lone pairs might interact with the edge of the aromatic core, providing additional stability (Figure S4) [77]. On the other hand, Val459 in *Pf*AM1 (PDB 3EBH) and Pro333 in *Hs*ERAP2 (PDB 4JBS) might be considered as straight and bulky, shortening the S1 pocket. In *Hs*LTA_4_H, Tyr267 (PDB 1HS6) in this position induces the formation of a narrow S1 tunnel. Moreover, domain III is missing in this latter enzyme and the presence of Tyr267, Lys565 and particularly Tyr378, which extends into the active site cavity pointing inward toward the Zn ion, reduced substantially the active site cavity volume (Figure S5). As a result of this spatial arrangement, steric clashes with the aromatic core of aminobenzosuberone might explain the very weak inhibitory activities of this scaffold on *Hs*LTA_4_H.

The aminobenzosuberone derivatives are markedly more potent on M1 aminopeptidases exhibiting a shallow S1 cavity with small to moderate size (*Ec*PepN, *Hs*APN, *Pf*AM1 and a borderline case for *Hs*IRAP). At the top of the elongated S1 subsite cylinder are located two or three cap residues (presented in bold type in Table 5 and depicted in stick in Figure 3a and Figure S3) from the C-terminal domain of the proteins. These residues are known to influence S1 subsite specificity [78,79]. Indeed, depending on the nature of the residues and through the conformational flexibility of their side chains, they can mitigate any potential unfavourable steric clashes and favour van der Waals, H-bond or electrostatic interactions with the substrates/inhibitors. Moreover, the closing of the S1 subsite induces the expulsion of water molecules from the active site into the bulk (entropically favourable) and may strengthen both van der Waals and electrostatic interactions. The analysis of superimposed structures showed that our aminobenzosuberone inhibitors were not expected to penetrate into the S1 cavity as deeply as other inhibitors (like bestatin) (Figure S4a and Figure S6), indicating they should therefore interact more efficiently with shallow S1 subsite. 

However, depending on the nature of the variable amino acid residue, the aminobenzosuberone core might slightly move upward to minimize any potential steric hindrance with this special ‘gatekeeper’ residue. Substituents on position-1 most likely induced steric conflicts with residues at the entrance of S1 cavities. More suitable substitutions in position-9 should offer new scopes for novel series of more potent and selective aminobenzosuberone derivatives (Figure S6).

### 3.3. S1′ Subsite

**Figure 4:**
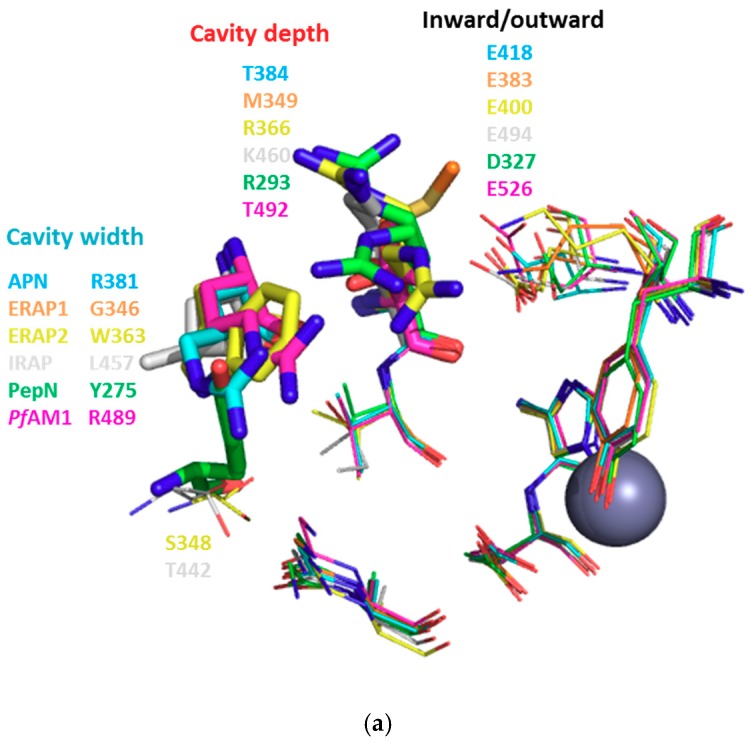

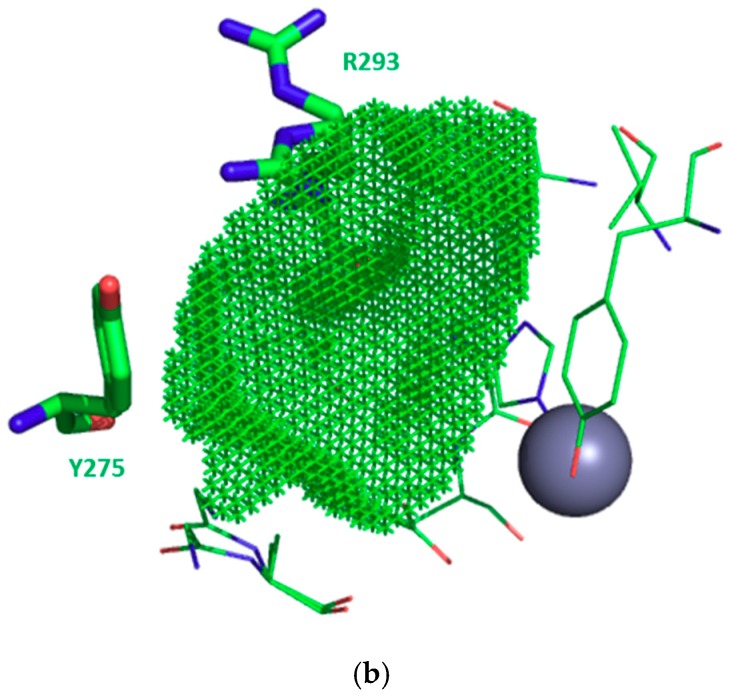
Diversity in the S1′ subsite of different M1 aminopeptidases. (**a**) Superimposition of the protein backbone of S1′ subsite of different M1 APs: *Hs*APN (cyan, PDB 4FYT), *Hs*ERAP1 (orange, PDB 2YD0), *Hs*ERAP2 (yellow, PDB 4JBS), *Hs*IRAP (white, PDB 5MJ6), *Ec*PepN (green, PDB 5MFS), *Pf*AM1 (magenta, PDB 3EBH). (**b**) Surface representation of the S1′ subsite of *Ec*PepN (green, PDB 5MFS). The catalytic zinc ion is shown as grey sphere. Residues involved in S1′ plasticity are shown in stick. Images generated with KVFinder plugin in PyMOL.

In contrast to S1, the S1′ subsite is an open cavity of 9 to 10 residues for all enzymes. For instance, in *Ec*PepN, Gly261, Ala262, Tyr275, Arg293, Val294, His297, Glu298, Val324, Asp327, Tyr381 compose this subsite. The careful analysis of the various 3D structures showed that only 2 to 3 residues (highlighted in blue and red in Table 6) seem to be involved in S1′ plasticity, thereby controlling the cavity width and depth (Figure 4a and Figure S7). Two amino acids, which are Tyr275 (located on the sidewall opposite to the catalytic zinc ion) and Arg293 (partially capping the S1′ cavity) for *Ec*PepN, vary in size and polarity within this enzyme family, as summarized in Table 6 and Figure 4a.

For example, *Pf*AM1 Arg489 is found more buried in the cavity than *Hs*APN Arg381, reducing thus slightly the width of S1′ subsite, whereas the *Ec*PepN Tyr275 and *Hs*APN Arg381 are positioned at a similar location, modifying thereby the local polarity. Also, the *Ec*PepN Arg293 side chain is located further inside the cavity reducing the cavity depth, whereas the less bulky and polar *Pf*AM1 Thr492 and *Hs*APN Thr384 occupy a similar position. Finally, local flexibility was observed within captured structures. Indeed, the side chain of residue *Hs*APN Arg381, *Hs*ERAP2 Arg366 and *Ec*PepN Arg293 can adopt different positions upon ligand binding. A similar observation might also be noticed for *Ec*PepN Asp327, this carboxylate side chain is either orientated in- or outside S1′ cavity, with the inside orientation potentially involved in further interactions as exemplified by *N. meningitides* alanyl aminopeptidase inhibition study [80]. In its close vicinity, a Lys residue is found in *Hs*ERAP1 and *Hs*ERAP2, Lys380 and Lys397, respectively, with their side chains extending in the cavity (residue in black bold in Figure S6). Developing interactions with this particular residue via *para*- or *meta*-substituted phenyl ring at position 4 of the aminobenzosuberone core should improve the selectivity and affinity for both members of this oxytocinase family.

All this information clarifies the inhibition observed for *Hs*APN and its microbial orthologues, *Ec*PepN and *Pf*AM1. Despite *Pf*AM1 S1′ cavity volume being slightly larger than *Hs*APN and *Ec*PepN (Table S4), the S1′ subsite cavity width of *Pf*AM1 is shorter, leading to a lack of binding affinity for the bulky ferrocenyl substituent of **21d** which cannot fit appropriately the *Pf*AM1 S1′ subsite (K_i_ > 50 µM). With regard to the SAR experimentally determined, it highlights the importance of the substitution in position-4 of the aminobenzosuberone core, which, according to our model, almost fully occupies this subsite in the evaluated enzymes. Most aminopeptidases can accommodate bromine or a phenyl group with an improved affinity of the inhibitor, when compared to the unsubstituted derivative. Nevertheless, a phenyl group is mostly preferred probably due to a possible π-stacking with *Ec*PepN His297, leading to the low K_i_ value of **21c** when compared to **21a** or **21b**.

### 3.4. Intra/Inter-Domain Movements

An important feature to be considered for M1 aminopeptidases molecular recognition is their inter- or intra-domain movements [20,21,22,23,24,25]. Various conformations have been captured during crystallization processes and open, intermediate and closed conformations have been determined for various given enzymes, suggesting that these dynamics are important for ligand binding and peptide hydrolysis [21,22,70,81]. A large inter-domains motion has been markedly observed for *Hs*ERAP1, associated with local changes for the highly conserved Tyr residue (Tyr438 for *Hs*ERAP1) in the active site (corresponding to the position Tyr381 of *Ec*PepN in Figure S1). In an “open” form of the protein, this residue undergoes a 6 Å shift away from the zinc ion, whereas in a closed form, this Tyr residue is proposed to both assist in the activation of the peptide bond and in the stabilization of the tetrahedral intermediate during the proteolytic catalytic cycle [21,22]. It has been shown, by site directed mutagenesis, that mutation of this Tyr residue into Phe completely abolished the enzyme activity [82,83]. 

As a result of this dynamic, the open form of the enzyme is likely catalytically inactive and a subsequent allosteric activation upon substrate binding might trigger a catalytically active closed form. This dynamic transition between those different states might help the substrates/products to bind to or to be released from the active site. In solution, a number of conformers might exist, from open to closed states, along with different position of the Tyr residue. We have previously suggested [60] that this Tyr residue probably activates the carbonyl function of the aminobenzosuberone towards a nucleophilic attack of the catalytic water to form a hydrated form of this ketone function and generates in situ a potent chelator of the zinc ion and a perfect mimic of the transition state. So, our low-molecular weight reversible inhibitors would be likely to bind to a closed form of *Hs*ERAP1 for optimum interactions. Interestingly, *Hs*IRAP was reported to undergo a similar conformational change with, this time, complete different orientation of the GAMEN motif upon inhibitor binding, leading to a closed conformation of *Hs*IRAP (PDB 5MJ6) and highlighting some significant structural plasticity of the GAMEN loop [24]. Since those enzymes in solution should exist in a conformational equilibrium between different forms (even so small angle X-ray scattering experiments have suggested that the *Hs*ERAP1 average structure in solution adopts an open conformation) [70,81], these processes could account for the moderate inhibition activity values observed with the *Hs*ERAP sub-family (IC_50_ = 1.6 to 150 µM) and, to a lesser extent, *Hs*IRAP (IC_50_ = 0.034 to 2.6 µM), indicating that the aminobenzosuberone scaffolds are not able to induce domain movements to a more closed form of the enzyme.

Intra-domain mobility is also to be considered. For example, besides a more compact folding, a particularity of the human and porcine APN structures determined up to now is the plasticity of the S1 subsite through conformational changes. It looks like a small/shallow hydrophobic pocket, compared to the deeper S1 subsites observed in other crystallized M1 aminopeptidases [20,25]. Indeed, *Hs*APN Phe896 which serves to cap the S1 pocket belongs to a flexible loop (as indicated by an average temperature factor of 25–35 Å^2^) of 8 residues (Y891GGGSFSF898) in domain IV and might act as a gatekeeper restricting access and closing the S1 subsite. Upon ligand binding, this S1 pocket might undergo a major conformational change via movement of the flexible 891–898 loop, revealing thus a deeper cylindrical hydrophobic S1 subsite [25]. The cavity volumes of the active site of *Hs*APN varied from 572.12 Å^3^ to 811.87 Å^3^ (Figure S8 and Table S2). Our series of compounds are very potent on this human enzyme, probably through interactions with the “Phe-In” conformation of this loop via the bromine substituent in position-1 of the aminobenzosuberone core and the aromatic ring of Phe896 (**8i**, K_i_ = 60 pM) [60,74]. These interactions might induce or stabilize the closure of domain IV, especially that of mammalian APN and hence a closed APN form, which is an important feature to block porcine coronavirus cell entry via its porcine APN receptor [23].

These different conformational dynamic processes seem to be an important aspect of catalytic regulation for some M1 enzymes, although it has yet to be more documented with a larger number of structures. These inter- or intra-domain movements are probably only one remarkable feature of aminopeptidase M1 dynamics, according to their biological functions and their cellular localization.

### 3.5. ADME-Tox Properties

To gain insight into the physicochemical and ADME-Tox properties of these aminobenzosuberone derivatives, several parameters were experimentally determined or computed (Table S5) [84]. The experimental logD_7.4_ values of the whole aminobenzosuberone series complied with Lipinski’s “rule of five” [85,86] and the calculated TPSA value is comprised between 44.71 and 68.10 Å^2^ for the ketone and hydrate forms, respectively. These compounds are predicted to penetrate the blood-brain barrier, although some possible P-glycoprotein (P-gp) efflux issues should be taken into consideration (predicted probability to be P-gp substrate is 0.5). They could be considered as valuable chemical tools to target M1 aminopeptidases implicated in CNS diseases, mostly involved in the brain renin-angiotensin system (*Hs*IRAP, *Hs*APN, *Hs*APA) [87,88,89] and in the pain management (*Hs*APN) [57,90]. However, the aminobenzosuberone scaffold requires careful optimization and increasing selectivity toward targeted M1 aminopeptidase versus *Hs*PSA, which plays major neuroprotective roles [91,92,93,94,95,96]. Analysis of the calculated probabilities of drug-drug interaction with different CYP450 isozymes shows that this series of molecules should inhibit CYP1A2 and to a lesser extent CYP2D6. In addition, the probability of toxicity is high and is mostly associated with cardiotoxicity with the inhibition of hERG channel and also hepatotoxicity. The uncertainties in the predicted outcomes should be taken into account and these potential toxicity liabilities should be investigated further. Thus, considering ADMET properties and inhibitory activities, the most promising compound for subsequent studies is the 1,4-dibromo derivative **21h**, which is also the most synthetically feasible molecule. However, according to the predictive method, decreasing the lipholicity (clogP ou logD_7.4_) by introducing heteroatoms into the phenyl ring of compound **21c** might also be a direction of modification since it should improve the ADME properties and have beneficial effects on toxicity. 

## 4. Materials and Methods 

### 4.1. General Information

Reactants were purchased from usual provider. Usual solvents were freshly distilled, dry MeOH distilled over Mg/MgI_2_, dry DME over Na and benzophenone, dry Et_2_O was distilled and stored over Na, DCM was distilled over P_2_O_5_ and stored over anhydrous K_2_CO_3_.

Flash chromatography: silica gel (Merck 60, 230–400 mesh). TLC: Al-roll silica gel (Merck 60, F254). Mp: Kofler hot bench, corrected. ^1^H- and ^13^C-NMR (400 MHz and 100.6 MHz resp.) spectra: Bruker Avance 400; δ in ppm and J in Hertz. 

All these racemic substituted 7-amino-5,7,8,9-tetrahydrobenzocyclohepten-6-one hydrochloride salts were already fully described in literature [58,60] and were resynthesized to determine their selectivity profiles. Their inhibitory activities against pAPN, hLTA_4_H and A*AP* were already reported [58,60].

### 4.2. General Procedure for Rubottom Oxidation

To an ice-cold mixture of water and acetone (70:35 mL) were added NaHCO_3_ (6.18 g, 20 eq.) and Oxone^®^ (11.3 g, 5 eq.). The suspension was stirred at 0 °C for 30 min and then a solution of silyl enol ether **15** (1.3 g, 1 eq.) in DCM (70 mL) was dropwise added. The mixture was warmed to r.t. and stirred for 3 h (TLC monitoring). Layers were separated and aqueous layer was extracted with DCM. Combined organic layers were washed with brine, dried on MgSO_4_, filtered and concentrated to give silyl-oxy ketone **16**, which was used without further purification.

### 4.3. General Procedure for Oxime Reduction

To a solution of hydroxy-oxime **17** (220 mg, 1 eq.) in methanol (13 mL) was added CoCl_2_·6H_2_O (388 mg, 2 eq.). The mixture was cooled to −30 °C and NaBH_4_ (462 mg, 15 eq.) was carefully added. The reaction was slowly warmed to r.t. and stirred for 2 h (TLC monitoring). The mixture was diluted with water and extracted by AcOEt. Organic layers were washed with brine, dried on MgSO_4_, filtered and concentrated to give amino-alcohol **18**, which was N-protected without further purification.

### 4.4. Production and Purification of Recombinant Aminopeptidases

#### 4.4.1. *Pf*AM1 and *Pf*AM17 

Genes encoding residues 192–1085 of native *Plasmodium falciparum* alanyl aminopeptidase *Pf*AM1 (PlasmoDB PF3D7_1311800) and residues 84–605 of native *Plasmodium falciparum* leucyl aminopeptidase *Pf*AM17 (PlasmoDB PF3D7_1446200) were synthetized by Genecust (Luxembourg), with the help of algorithms developed for optimizing, DNA sequences to improve proteins expression in *Escherichia coli* [97]. *i*Synthetic genes were cloned into the T7 expression pET45b(+) vector (Novagen), which appended a *N*-terminal hexahistidine tag (*KnpI* and *SalI* sites for PfA-M1–*BamHI* and *SalI* sites for PfA-M17). 

*Escherichia coli* Rosetta 2 (DE3) bacteria (Novagen) were transformed with these recombinant plasmids, after their validation by Sanger sequencing (Beckman Coulter Genomics). Bacterial cultures were grown in auto-induced LB medium (Merck) supplemented with carbenicillin (50 µg/mL) and chloramphenicol (34 µg/mL), during 24 h at 25 °C, prior to bacterial extract preparations with BugBuster^TM^ (Novagen, Darmstadt, Germany). The clarified lysates were loaded onto Ni_2_-charged HisTrap column (GE Healthcare) equilibrated in 20 mM imidazole phosphate buffer and washed in the same buffer. Bound recombinant proteins were then eluted in 80 mM imidazole for *Pf*AM1 and 200 mM imidazole for *Pf*AM17, in phosphate buffer. Eluted fractions were finally purified by size exclusion chromatography on a Superdex 200 10 300 (equilibrated with Tris HCl 50 mM, NaCl 200 mM, ZnCl_2_ 10 µM, pH 7.4 for *Pf*AM1 and with Hepes 50 mM, NaCl 300 mM, ZnCl_2_ 10 µM, 5% glycerol, pH 8.5 for *Pf*AM17) using an Äkta purifier chromatography system (GE Healthcare Life Science, Little Chalfont, England). 

#### 4.4.2. *Hs*ERAP1, *Hs*ERAP2 and *Hs*IRAP

The expression and purification of recombinant human endoplasmic reticulum aminopeptidase 1 (*Hs*ERAP1), endoplasmic reticulum aminopeptidase 2 (*Hs*ERAP2) and insulin-regulated aminopeptidase (*Hs*IRAP) have been described before [79,98,99]. Briefly, recombinant baculovirus containing the gene for each enzyme was produced in sf9 cells according to the manufacturer’s instructions (Bac-to-Bac baculovirus expression system, Invitrogen). Recombinant proteins were expressed in Hi5 cells after infection with the appropriate recombinant baculovirus and purified by NiNTA affinity chromatography as previously described (PMID: 21314638). Proteins were aliquoted and stored at −80 °C in a buffer containing 10 mM Hepes pH 7.0, 100 mM NaCl, 10% glycerol, until needed.

#### 4.4.3. *Ec*PepN and *Hs*PSA

Both the enzymes were purified as reported earlier [73,100]. 

### 4.5. Enzymatic Assays and Kinetic Analysis

#### 4.5.1. *Pf*AM1 and *Pf*AM17

Tests were carried out on HP/Agilent UV-Visible diode array spectrophotometer 8453 (HP/Agilent, Santa Clara, CA, USA), at 30 °C in Tris HCl 50 mM pH 7.4 for r*Pf*AM1 and pH 8 for r*Pf*AM17, with a final DMSO concentration of 1%. Enzyme activities of r*Pf*AM1 (10 nM) and r*Pf*AM17 (60 nM) were determined by continuously measuring the release of para-nitroaniline at 405 nm with alanine para-nitroanilide as substrate for r*Pf*AM1 (Km = 1.5 mM) and leucine para-nitroanilide for r*Pf*AM17 (Km = 0.5 mM). After 10 min of incubation, CI_50_ measurements were conducted during 30 min for r*Pf*AM1 and 3 h for r*Pf*AM17. Initial velocities were measured with increasing concentrations of inhibitors and K_i_ values were determined by Dixon plots in triplicate.

#### 4.5.2. LTA_4_H

5 µg of rLTA_4_H (Sigma) were used to measure the activity of this protein through the release of alanine para-nitroanilide at 405 nm (Km = 2 mM). Experiments were performed on HP/Agilent UV-Visible diode array spectrophotometer 8453 at 30 °C, during 30 min, in Tris HCl 10 mM, KCl 0.1 mM, pH 7.5 to obtain IC_50_ values.

#### 4.5.3. EcPepN and *Hs*PSA 

Enzyme activity assays were carried out in 10 mM Tris-HCl buffer at pH 7.5 with 150 mM NaCl, enzyme, 100 µM substrate (Leu-*p*NA) in a 100 μL reaction at 37 °C. The absorbance was read at 405 nm. Concentrations ranging from 1 nM to 100 μM of inhibitors (dissolved in DMSO) were incubated with each of the enzymes (75 µM of *Ec*PepN, 120 µM of *Hs*PSA) for 30 min at 37 °C in a 96 well plate. After addition of the substrate, immediate increase in absorption was measured. All inhibition assays were performed in triplicates and initial velocities were measured. Percentage inhibition and log values of inhibitor concentrations were plotted using SigmaPlot to determine IC_50_ values. K_i_ values were determined by the Dixon method. The assay consists of reaction buffer, enzyme and varied concentrations of Leu-pNA (25, 50, 100, 200 and 400 µM). Based on the IC_50_ values, at each substrate concentration, inhibitor concentration was varied in the range of 50 nM to 500 nM or 0.5 µM to 25 µM. Data were plotted as 1/rate versus inhibitor concentration for each substrate concentration and a linear fit was calculated by non-linear regression using SigmaPlot12.5. All the reactions were performed in triplicates, SD values were reported [73].

#### 4.5.4. *Hs*ERAP1, *Hs*ERAP2 and *Hs*IRAP

The enzymatic activity of *Hs*ERAP1, *Hs*ERAP2 and *Hs*IRAP was determined by following the time-dependent increase in fluorescence at 460 nm (excitation: 380 nm) of the fluorigenic substrates L-Leucine-7-amido-4-methyl coumarin (L-AMC; Sigma, Darmstatd, Germany) for *Hs*ERAP1 and *Hs*IRAP and L-arginyl-7-amido-4-methyl coumarin (R-AMC; Sigma) for *Hs*ERAP2. Measurements were performed on a 96-well plate (150 μL total volume in each well) on a TECAN infinite M200 microplate fluorescence reader. 30 nM of *Hs*ERAP1, 6 nM of *Hs*ERAP2 or 6 nM of *Hs*IRAP was added in each well, along with 50 μM of substrate and varied concentrations of compound (within the range of 1 nM to 200 μM). The reaction was followed for 10 min at room temperature and the remaining enzymatic activity was calculated by measuring the slope of the time course. For calculation of the in vitro IC_50_ values, experimental data were fit to the following equation using the GraphPad Prism software package:

Y = Bottom + (Top − Bottom)/(1 + 10^((LogIC_50_ − X) * HillSlope)) (1)

where Y is the enzymatic activity and X the inhibitor concentration. 

### 4.6. Measurement of Octanol/Water Partition Coefficient (logD_7.4_) 

The lipophilicity (logD_7.4_ values) of the amino-benzosuberones was determined by reversed-phase HPLC on a C-18 column (Eclipse XDB-C18, 5 µm, 4.6 × 150 mm, from Agilent, France) according to the method previously described by Minick and Pomper [101,102]. Measurement of the chromatographic capacity factors (k′) for each compound was done at various concentrations in the range of 90–60% methanol (containing 0.25% octanol) and an aqueous phase consisting of 0.15% n-decylamine in 0.02 M MOPS (3-(*N*-morpholino)propanesulfonic acid) buffer pH 7.4 (prepared in 1-octanol-satured water). The flow was 0.9 mL/min. The aminobenzosuberone derivatives were dissolved at 0.4 µM in methanol. The standards were also dissolved in methanol. Column void volume was estimated from the retention time of uracil, which was included as a non-retained internal reference with each injection. The capacity factors (k′) are extrapolated to 100% of the aqueous component given the value of k′w. The logD_7.4_ is then obtained by the formula:

logD_7.4_ = 0.13418 + 0.98452k′w (2)

Positive control with β-oestradiol, experimental logD_7.4_ 3.043 ± 0.093 (*n* = 9), literature value 3.261.

### 4.7. In Silico Prediction of ADMET Properties

The study of ADMET properties was carried out on the website http://admet.scbdd.com [84]. 

## 5. Conclusions

M1 family aminopeptidases have broad and overlapping substrate specificity; hence small-molecule inhibitors may not always be specific, especially regarding their selectivity toward bimetallic enzymes. The aminobenzosuberone scaffold demonstrated exclusive selectivity for the monometallic M1 aminopeptidase family with particular potent inhibitory activities against mammalian APN and its microbial orthologues *Ec*PepN and *Pf*AM1 (K_i_ values in the nanomolar range). It retains interesting potency on *Hs*PSA and also on *Hs*IRAP (K_i_ values in the nano- to sub-micromolar range) but it is seemingly less active on both oxytocinase enzymes *Hs*ERAP1/2 (IC_50_ values in the micromolar range) and totally inactive against *Hs*LTA_4_H. 

This promising scaffold represents an attractive new class of potent antimalarial compounds targeting *Pf*AM1 [69], a crucial enzyme involved in at least the last committed step of haemoglobin degradation by *Plasmodium falciparum* [17,31,35]. In addition, growing evidences highlight the crucial role played by mammalian aminopeptidases in a great variety of cancer types, especially *Hs*APN and *Hs*ERAP1/2 via their proteolytic activity or their ability to modulate protein-protein interactions [103]. The aminobenzosuberone core is an attractive starting point to design triple inhibitor of all these three enzymes, acting by interfering with endothelial cell morphogenesis and cell motility [61] and by modulating antigen processing to trigger cancer immunotherapy [14,15,16]. 

The next challenge is to rationally design selective inhibitors for individual M1 aminopeptidase, to study their biological roles and precise their functions, or to avoid any potential adverse effects following in vivo treatment with aminobenzosuberone derivatives by targeting other members of this diverse family. To achieve this goal, the design process efforts should take into account the plasticity of the active site and the conformational dynamics of these M1 aminopeptidases. Interesting hints suggest deeper interactions into the S1 subsite through a substitution on position-9 of our scaffold should offer new opportunities to improve both activity and selectivity. Another approach for achieving selectivity is to look for the cellular/subcellular localization and/or tissue distribution of the targeted aminopeptidase, to design targeted-prodrug to improve site-specific drug delivery. These different strategies are currently under investigation and will be reported in due course.

## Figures and Tables

**Table 1:** Current established biological functions for representative members of the M1 aminopeptidases [1,10,11].

Organism	M1 Family	Biological Roles	Associated Diseases
*H. sapiens*	APN	Metabolism of regulatory peptides of diverse cell types Processing of peptide hormones (Angiotensin III and IV, neuropeptides and chemokines Regulation of angiogenesis, cell motility, cell-cell adhesion Coronavirus receptor	Pain sensation, Inflammatory diseases, cancer & upper respiratory tract infections
	APA	Activation or inactivation of various components of the angiotensin system	Hypertension
	LTA_4_H	Biosynthesis of the proinflammatory mediator LTB-4	Inflammatory & allergic diseases
	TRHDE	Inactivation of Thyrotropin Releasing Hormone (brain)	
	PSA	Regulation of neuropeptide activity, digestion of polyQ peptides Antigen processing pathway for MHC class I molecules	hematologic cancer Impedes the development of neuropathology
	IRAP	Peptide hormone degradation (oxytocin, vasopressin, angiotensin III) Maintain homeostasis during pregnancy Inactivation of neuronal peptide (enkephalin, dynorphin) Generation of antigenic peptides in dendritic cells [12]	cognitive impairment (Alzheimer’s disease, head trauma, cerebral ischemia) Inflammatory autoimmunity [13]
	APB	Biosynthesis of the proinflammatory mediator LTB-4	
	ERAP1	Regulation of blood pressure Antigen processing pathway for MHC class I molecules	Autoimmune diseases (ankylosing spondylitis, psoriasis, type 1 diabetes, Crohn…) Cancer [14,15,16]
	ERAP2	Antigen processing pathway for MHC class I molecules	
	APQ	Placentation: regulation of biological activity of key peptides at the embryo-maternal interface	Pre-eclampsia
	APO	Activation or inactivation of various components of the angiotensin system	
*P. falciparum*	*Pf*AM1	Catabolism of host haemoglobin in the food vacuole of Plasmodium	Malaria [17]
*E. coli*	PepN	Cytosolic peptide catabolism and adaptation to nutritional downshift and high temperature stress	

**Table 2:** Substrate specificity: preferred *N*-terminus amino-acid for a selection of M1 aminopeptidases [1].

M1 Aminopeptidase	Substrate Specificity: Favoured *N*-Terminus Amino-Acid
**APN**	Ala, Phe, Tyr, Leu, most of aa including Pro (slow)
**APA**	Glu and to a lesser extent Asp
**LTA_4_H**	Ala, Arg, Leu, Pro
**TRHDE**	pGlu (pyroglutamyl)
**PSA**	Ala, Leu, Lys, most of aa (except Gly and Pro)
**IRAP**	Cys, Leu, Arg, Ala and most of aa (except Asp, Glu) including cyclic peptides [27]
**APB**	Arg, Lys
**ERAP1**	Leu and most of aa including Met, Cys, Phe
**ERAP2**	Arg, Lys
**RNPL1**	Ala, Lys, Ser, Ile, Met, most of aa
**APQ**	Leu, Arg, Lys, Met, most of aa
**APO**	Arg and to a lesser extent Asn
***Pf*AM1**	Ala, Leu, Lys, Arg and most of aa (except Pro, Asp, Glu)
**PepN**	Arg, Ala, most of aa including Pro

**Table 3:** K_i_ or * IC_50_ values in micromoles reported in literature for representative M1 AP’s inhibitors.

Compound	M1 Aminopeptidases ‘Monometallic’										M17 & M28 Aminopeptidases ‘Bimetallic’		
	*Hs*APA	*Hs*APB	mAPN	*Hs*ERAP1	*Hs*ERAP2	*Hs*IRAP	*Hs*LTA_4_H	*Hs*PSA	*Ec*PepN	*Pf*AM1	A*AP*	mLAPc	*Pf*AM17
**1**	NI [38]	0.014 [39]–6.0 [38]	0.3 * [37]–89.0 [40]	11.2 [41]			0.2 * [37] 0.5 [42]	0.35 * [37]	7.3 * [43]	0.19 [44]	0.0016 [42]	0.0006 [38]–0.020 [39]	0.025 [45]
**2**	-	-	0.002 [46]	-	-	-	-	-	-	0.079 [47]	-	0.066 [33]	0.013 [45]
**3** [48]				0.043 *	0.037 *	0.002 *							
**4** [49]			>100					1.79 *	>100	>100			
**5** [38]	>1000	>1000	0.02									3.0	
**6** [50]	0.14		0.12										
**7** [37] **8** [37]	-	>1 *	0.22 *	>5 *	-	-	>10 * 0.008 *	0.15 * 0.85 *	-	6 [36]	-	0.1 * 0.03 *	0.079 [36] -
**9**	>100 [40]	>100 [51]	0.3 [52]–2.0 * [40]									2.6 * [51]	
**10** [35,53]			1.37 *							0.006 *			
**11** [54]				0.92 *	1.6 *	0.105 *							
**12** [55]			>100					0.8 *					
**13** [56]						0.03							

M1 monometallic enzymes: human aminopeptidase A (*Hs*APA), human arginyl aminopeptidase B (*Hs*APB), mammalian aminopeptidase N (mAPN), human endoplasmic reticulum aminopeptidase 1 (*Hs*ERAP1), human endoplasmic reticulum aminopeptidase 2 (*Hs*ERAP2), human Leucyl-cystinyl aminopeptidase (*Hs*IRAP), human leukotriene A_4_ hydrolase (*Hs*LTA_4_H) and human puromycine sensitive aminopeptidase (*Hs*PSA); *E. coli* (*Ec*PepN) and *P. falciparum* (*Pf*AM1) M1 aminopeptidases; representative members of the bimetallic aminopetidases from *A. proteolytica* (A*AP*), mammalian cytosolic leucine aminopeptidase (mLAPc) and *P. falciparum* M17 aminopeptidase (*Pf*AM17). NI, no inhibition. * IC_50_.

**Table 4:** Inhibition of selected aminopeptidases by racemic substituted 7-amino-5,7,8,9-tetrahydrobenzocyclohepten-6-one hydrochloride salts.

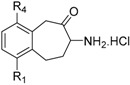	R_1_	R_4_	M1 Aminopeptidases ‘Monometallic’								M17 & M28 Aminopeptidases ‘Bimetallic’	
			Papn ^k^ [58]	r*Pf*AM1 ^l^	R*Ec*PepN ^m^	r*Hs*ERAP1 ^n^ *	r*Hs*ERAP2 ^o^ *	r*Hs*PSA ^p^	r*Hs*IRAP ^q^ *	r*Hs*LTA_4_H ^s^ [58]	*rPf*AM17 ^t^	A*AP* ^u^ [58]
**21a**	H	H	1	15 ± 0.07	50 ± 3.0	-	-	-	-	>100	-	>100
**21b**	H	Br	0.04	0.04 ± 0.009	1.07 ± 0.11	120 ± 11	49 ± 3.8	1.63 ± 0.08	1.0 ± 0.08	>100	>100	>100
**21c**	H	Ph	0.007	0.05 ± 0.005 [69]	0.16 ± 0.012	63 ± 3	18 ± 1.3	0.05 ± 0.004	2.4 ± 0.3	19	>100 [69]	28
**21d**	H	Fc	0.004 [60]	>50	0.06 ± 0.008	-	-	-	-	-	-	-
**21e**	Br	H	0.02	1 ± 0.04	4.59 ± 0.17	56 ± 7	104 ± 7	1.07 ± 0.063	2.6 ± 0.2	>100	>100	>100
**21f**	Ph	H	0.25	2 ± 0.07	-	137 ± 5	>200	-	2.2 ± 0.13	>100	>100	>100
**21g**	Benzo [1,2]		0.04	15 ± 1	-	-	-	-	-	>100	-	>100
**21h**	Br	Br	0.006	0.005 ± 0.001	-	5.5 ± 1.1	2.8 ± 0.3	-	0.034 ± 0.001	>100	-	>100
**21i**	Br	Ph	0.00006	0.03 ± 0.005	0.05 ± 0.003	1.6 ± 0.1	0.39 ± 0.013	0.021 ± 0.003	0.12 ± 0.015	68% ^#^	-	39
**21j**	Ph	Br	0.07	4 ± 0.7	-	-	-	-	-	54% ^#^	*-*	>100

K_i_ values (µM) determined from Dixon plots or * IC_50_ values (µM) of compounds **21a**–**j** with, **k**—pAPN from porcine kidney, **l**—*P. falciparum* r*Pf*AM1. **m**—*E. coli* r*Ec*PepN, **n**—human endoplasmic reticulum aminopeptidase 1 (r*Hs*ERAP1), **o**—human endoplasmic reticulum aminopeptidase 2 (r*Hs*ERAP2), **p**—human puromycine sensitive aminopeptidase (r*Hs*PSA), **q**—human Leucyl-cystinyl aminopeptidase (r*Hs*IRAP), **s**—human leukotriene A_4_ hydrolase (r*Hs*LTA_4_H), **t**—*P. falciparum* r*Pf*AM17 and **u**—aminopeptidase from *A. proteolytica* (A*AP*). r stands for recombinant and Fc for Ferrocenyl. # % of inhibition determined at 100 µM. Inhibition values for pAPN, *Hs*LTA_4_H and A*AP* were already reported in literature [58,60].

**Table 5:** Amino acid composition of the S1 subsite of selected M1 APs.

M1 AP	PDB	Amino Acid Composition of the S1 Subsite																			
*Hs*APN	4FYT		Q_211_	Q_213_				N_350_	**A_351_**	G_352_	A_353_	M_354_	E_355_	H_392_	E_411_		F_472_	Y_477_			**F_896_**
	4FYR		Q_211_	Q_213_	A_214_	D_216_		N_350_	**A_351_**	G_352_	A_353_	M_354_	E_355_	H_392_	E_411_	**S_469_**	F_472_	Y_477_	S_895_		**S_897_**
*Hs*ERAP1	2YD0	H_160_	Q_181_	E_183_	P_184_	A_186_	F_314_	Q_315_	**S_316_**	G_317_	A_318_	M_319_	E_320_	H_357_	E_376_	**R_430_**	F_433_	Y_438_	E_865_	S_868_	**S_869_**
*Hs*ERAP2	4JBS	E_177_	D_198_	E_200_	P_201_	Q_203_	F_331_	A_332_	**P_333_**	G_334_	A_335_	M_336_	E_337_	H_374_	E_393_	**Q_447_**	F_450_	Y_455_	**Y_892_**		**R_895_**
*Hs*IRAP	5MJ6	Y_272_	Q_293_	E_295_	P_296_		F_425_	E_426_	**A_427_**	G_428_	A_429_	M_430_	E_431_	H_468_	E_487_	**E_541_**	F_544_	Y_549_	P_957_		**Y_961_**
*Hs*LTA_4_H	1HS6		Q_134_	Q_136_	A_137_				**Y_267_**	G_268_	G_269_	M_270_	E_271_	H_299_	E_318_	D_375_	Y_378_	Y_383_			
*Ec*PepN	5MFS		Q_119_	E_121_	A_122_				**M_260_**	G_261_	A_262_	M_263_	E_264_	H_301_	E_320_		Y_376_	Y_381_			**R_825_**
	2DQM		Q_119_	E_121_	A_122_			N_259_	**M_260_**	G_261_	A_262_	M_263_	E_264_	H_301_	E_320_	**N_373_**	Y_376_	Y_381_	Q_821_		**R_825_**
*Pf*AM1	3EBH	T_305_	Q_317_	E_319_	A_320_	T_321_	F_457_	N_458_	**V_459_**	G_460_	A_461_	M_462_	E_463_	H_500_	E_519_	**E_572_**	Y_575_	Y_580_			**M_1034_**

In red, the variable residue determined for the corresponding pdb entries. In blue, residue situated at the top of S1 subsite and involved in the capping of the pocket.

**Table 6:** S1′ subsite composition.

M1 AP	Amino Acid Composition of the S1′ Subsite												
*Hs*APN	G_352_	A_353_		**R_381_**	**T_384_**	V_385_	H_388_	E_389_	S_415_	**E_418_**	Y_477_		
*Hs*ERAP1	G_317_	A_318_		**G_346_**	**M_349_**	T_350_	H_353_	E_354_	K_380_	**E_383_**	Y_438_		
*Hs*ERAP2	G_334_	A_335_	S_348_	**W_363_**	**R_366_**	V_367_	H_370_	E_371_	K_397_	**E_400_**	Y_455_		
*Hs*IRAP	G_428_	A_429_	T_442_	**L_457_**	**K_460_**	I_461_	H_464_	E_465_	T_491_	**E_494_**	Y_549_		
*Hs*LTA_4_H	G_268_	G_269_			**N_291_**	V_292_	H_295_	E_296_	V_322_	**E_325_**	Y_383_	**R_563_**	**K_565_**
*Ec*PepN	G_261_	A_262_		**Y_275_**	**R_293_**	V_294_	H_297_	E_298_	V_324_	**D_327_**	Y_381_		
*Pf*AM1	G_460_	A_461_		**R_489_**	**T_492_**	V_493_	H_496_	E_497_	V_523_	**E_526_**	Y_580_		

Highlighted residues are involved in S1′ site. In blue, residues which reduce the cavity width; in red, residues which modulate the cavity depth and in bold, flexible amino acid side chains pointing either inward or outward the active site.

## Supplementary Materials

The following are available online. Figure S1: Conserved active site residues involved in substrate recognition and catalysis, Table S1: Human proteinase selectivity profiles of **21a**–**c** and **21i**, Table S2: Cavity volume of the active site of “closed” conformation of M1 APs, Figure S2: Surface representation of the active site of studied M1 Aminopeptidases, Figure S3: Surface representation of the S1 subsite of different M1 aminopeptidases, Table S3: Cavity volume of the S1 subsite of “closed” conformation of M1 APs, Figure S4: Local movement of Met260 in the active site of *Ec*PepN, Figure S5: Superimposition of the protein backbone of the aminobenzosuberone **21c** in *Ec*PepN complex (green, PDB 5MFS) with *Hs*LTA_4_H (slate blue, PDB 1HQ6), Figure S6: Superimposition of the protein backbone of the aminobenzosuberone **21c** in *Ec*PepN complex (green, PDB 5MFS) with different M1 aminopeptidases, Figure S7: Surface representation of the S1′ subsite of different M1 aminopeptidases, Table S4: Cavity volume of the S1′ subsite of “closed” conformation of M1 APs, Figure S8: Intra-domain movement in the active site of *Hs*APN, Table S5: Determination and prediction of various ADME-Tox properties of substituted 7-amino-5,7,8,9-tetrahydrobenzocyclohepten-6-one hydrochloride salts.
