# Supplementary material for: Aminobenzosuberone Scaffold as a Modular Chemical Tool for the Inhibition of Therapeutically Relevant M1 Aminopeptidases
Source: Molecules. 2018 Oct 11;23(10):2607. doi: 10.3390/molecules23102607 (PMC6222927; doi:10.3390/molecules23102607)
Supplement: Supplementary file 1 [file molecules-23-02607-s001.pdf]

molecules

Supplementary Materials

Aminobenzosuberone scaffold as a modular chemical tool for the inhibition of therapeutically relevant M1 aminopeptidases

Emmanuel Salomon [1,†], Marjorie Schmitt [1,†], Anil Kumar Marapaka [2,3,†], Athanasios Stamogiannos [4,†], Germain Revelant [1], Céline Schmitt [1], Sarah Alavi [1], Isabelle Florent [5], Anthony Addlagatta [2,3], Efstratios Stratikos [4], Céline Tarnusa [1,‡] and Sébastien Albrecht [1,*]

[1] Laboratoire d'Innovation Moléculaire et Applications, Université de Haute-Alsace, Université de Strasbourg, CNRS, 68093 Mulhouse, France; emmanuel.salomon@uha.fr (E.S.); marjorie.schmitt@uha.fr (M.S.); germain.revelant@hotmail.fr (G.R.); celine.schmitt85@gmail.com (C.S.); SALAVI@esta-groupe.fr (S.A.); celine.tarnus@uha.fr (C.T.)
[2] Centre for Chemical Biology, CSIR-Indian Institute of Chemical Technology, Hyderabad-500007, Telangana, India; anilmarapaka@gmail.com (A.K.M.); anthonyaddlagatta@gmail.com (A.A.)
[3] Academy of Scientific and Innovative Research (AcSIR), Rafi Marg, New Dehli-110001, India
[4] Protein Chemistry Laboratory, IRRP, National Centre for Scientific Research Demokritos, Agia Paraskevi, Athens 15310, Greece; a.stamogiannos@gmail.com (A.S.); stratikos@gmail.com (E.S.)
[5] Molécules de Communication et Adaptation des Micro-organismes, Muséum National d'Histoire Naturelle, CNRS, 75231 Paris, France; isabelle.florent@mnhn.fr
* Correspondence: sebastien.albrecht@uha.fr; Tel.: +33-389-336-714
† These authors contributed equally to this work.
‡ Current address: Laboratoire Vigne, Biotechnologies et Environnement, Université de Haute-Alsace, Université de Strasbourg, 68008 Colmar, France

List of Supplementary Materials

Figure S1. Conserved active site residues involved in substrate recognition and catalysis

Table S1. Human proteinase selectivity profiles of **21a–c** and **21i**

Table S2. Cavity volume of the active site of "closed" conformation of M1 APs

Figure S2. Surface representation of the active site of studied M1 Aminopeptidases

Figure S3. Surface representation of the S1 subsite of different M1 aminopeptidases

Table S3. Cavity volume of the S1 subsite of "closed" conformation of M1 APs

Figure S4. Local movement of Met260 in the active site of *Ec*PepN

Figure S5. Superimposition of the protein backbone of the aminobenzosuberone **21c** in *Ec*PepN complex (green, PDB 5MFS) with *Hs*LTA$_4$H (slate blue, PDB 1HQ6)

Figure S6. Superimposition of the protein backbone of the aminobenzosuberone **21c** in *Ec*PepN complex (green, PDB 5MFS) with different M1 aminopeptidases

Figure S7. Surface representation of the S1' subsite of different M1 aminopeptidases

Table S4. Cavity volume of the S1′ subsite of "closed" conformation of M1 APs

Figure S8. Intra-domain movement in the active site of *Hs*APN

Table S5. Determination and prediction of various ADME-Tox properties of substituted 7-amino-5,7,8,9-tetrahydrobenzocyclohepten-6-one hydrochloride salts

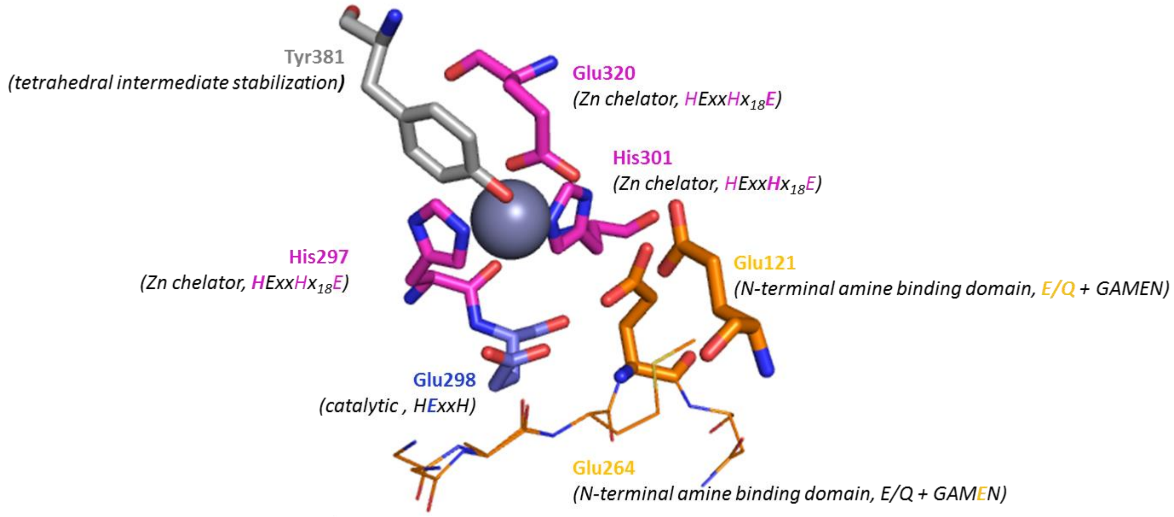

Figure S1. Conserved active site residues involved in substrate recognition and catalysis. Numbering corresponds to *Ec*PepN (PDB 5MFS). The zinc ion is shown as a grey sphere, key residues are represented in stick and part of the GAMEN domain is depicted in line (prepared using PyMOL Molecular Graphics System).

Table S1. Human proteinase selectivity profiles of **21a–c** and **21i**

Protease class	Enzyme Name	IC_{50} (μM)
aspartic	Pepsin A (pH 3.5)	> 30
cysteine	Caspase3	> 30
	CathepsinK	> 30
	CathepsinG	> 30
metallo	Aminopeptidase P2	> 30
	Neprilysin	> 30
	Angiotensin converting enzyme1	> 30
	MMP01	> 30
	MMP02	> 30
	MMP03	> 30
	MMP07	> 30
	MMP08	> 30
	MMP09	> 30
	MMP12	> 30
	MMP13	> 30
	MMP14	> 30
serine	DPP4	> 30
	Chymase	> 30
	Chymotrypsin	> 30
	FactorXa	> 30
	Kallikrein5	> 30
	Kallikrein7	> 30

	Neutrophil elastase	> 30
	Cationic Trypsin	> 30
	Thrombin	> 30

Private Partner *in vitro* screening data. Personal data.

Table S2. Cavity volume of the active site of "closed" conformation of M1 APs

« closed » conformation of M1 AP		ligand	PDB entry	Volume (Å^3)
*Hs*APN	intradomain movement : motion of Y891GGGSFSF898 loop	AMA	4FYT	572.12
		**1**	4FYR	811.87
*Hs*ERAP1		**1**	2YD0	875.37
*Hs*ERAP2	local movement : motion of R366 in S1′	APA	5AB0	872.25
		PPT1	4JBS	852.62
*Hs*IRAP		PPT2	5MJ6	894.12
*Hs*LTA$_4$H		**1**	1HS6	694.13
*Ec*PepN	local movement: motion of M260 residue in S1 motion of R293 residue in S1′	**21a**	5MFR	467.88
		**21c**	5MFS	531.25
		**21i**	5MFT	484.50
		**1**	2DQM	638.5
*Pf*AM1		**1**	3EBH	727.75

The cavity volume was calculated using KVFinder plugin in PyMOL. Ligand abbreviations stand for: AMA amastatin, APA aminophosphonic acid, PPT1/2 phosphinic pseudotripeptides.

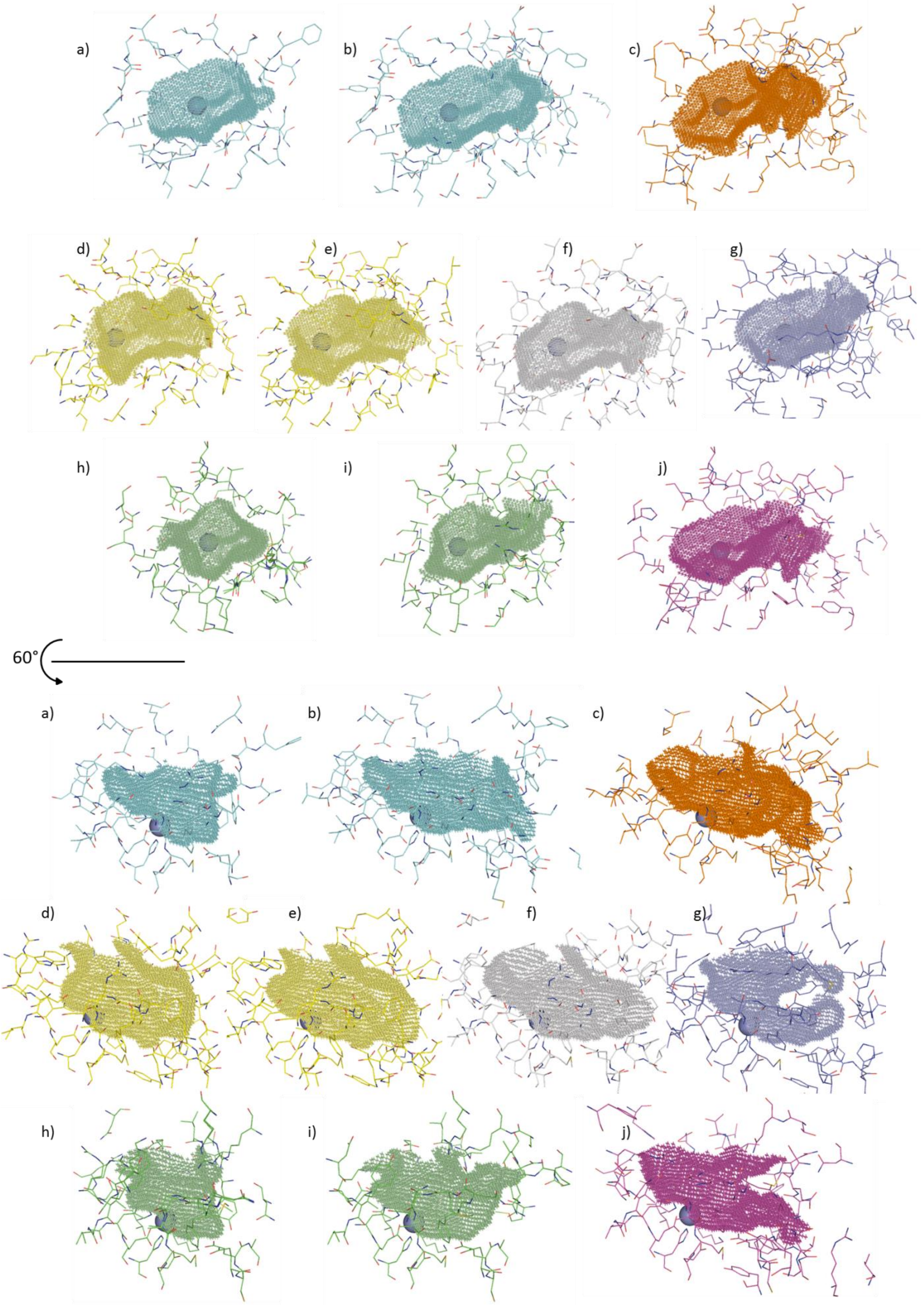

Figure S2. Surface representation of the active site of studied M1 Aminopeptidases. a) *Hs*APN (cyan, PDB 4FYT, cavity volume 572.12 Å^3), b) *Hs*APN (cyan, PDB 4FYR, cavity volume 811.87 Å^3), c) *Hs*ERAP1 (orange, PDB 2YD0, cavity volume 875.37 Å^3), d) *Hs*ERAP2 (yellow, PDB 4JBS, cavity volume 852.62 Å^3), e) *Hs*ERAP2 (yellow, PDB 5AB0, cavity volume 872.25 Å^3), f) *Hs*IRAP (white, PDB

5MJ6, cavity volume 894.12 Å^3), g) *Hs*LTA$_4$H (blue, PDB 1HS6, cavity volume 694.13 Å^3), h) *Ec*PepN (green, PDB 5MFS, cavity volume 531.25 Å^3), i) *Ec*PepN (green, PDB 2DQM, cavity volume 638.5 Å^3), j) *Pf*AM1 (magenta, PDB 3EBH, cavity volume 727.75 Å^3). The active site cavity was computed by KVFinder plugin in PyMOL. The active site residues are represented in line and zinc ion is shown as grey sphere.

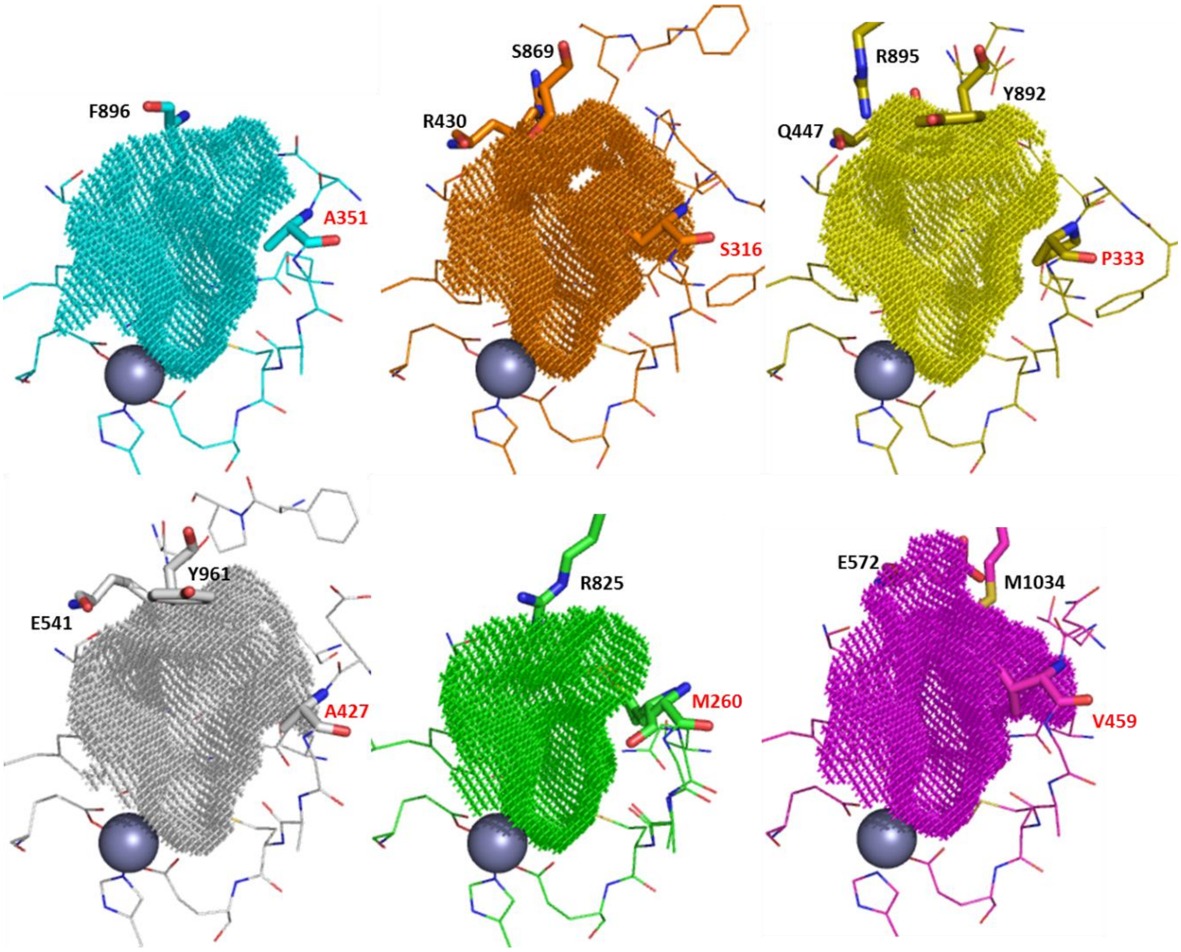

Figure S3. Surface representation of the S1 subsite of different M1 aminopeptidases. *Hs*APN (cyan, PDB 4FYT), *Hs*ERAP1 (orange, PDB 2YD0), *Hs*ERAP2 (yellow, PDB 4JBS), *Hs*IRAP (white, PDB 5MJ6), *Ec*PepN (green, PDB 5MFS), *Pf*AM1 (magenta, PDB 3EBH). The catalytic zinc ion is shown as grey sphere. Residues involved in S1 plasticity are shown in stick. In red, variable residues at the entrance of the cavity; in black, residues capping the pocket. Images generated with KVFinder plugin in PyMOL

Table S3. Cavity volume of the S1 subsite of "closed" conformation of M1 APs

« closed » conformation of M1 AP		ligand	PDB entry	Volume (Å^3)
*Hs*APN	intradomain movement : motion of Y891GGGSFSF898 loop	AMA	4FYT	377.22
		**1**	4FYR	641.41
*Hs*ERAP1		**1**	2YD0	610.88
*Hs*ERAP2		APA	5AB0	651.00
		PPT1	4JBS	676.22
*Hs*IRAP		PPT2	5MJ6	599.87

*Ec*PepN	local movement: motion of M260 residue in S1	**21c**	5MFS	289.79
		**1**	2DQM	442.24
*Pf*AM1		**1**	3EBH	500.54

The cavity volume was calculated using KVFinder plugin in PyMOL.; ligand abbreviations stand for : AMA amastatin, APA aminophosphonic acid, PPT1/2 phosphinic pseudotripeptides. In blue, residues which reduce the cavity width; in red, residues which modulate the cavity depth.

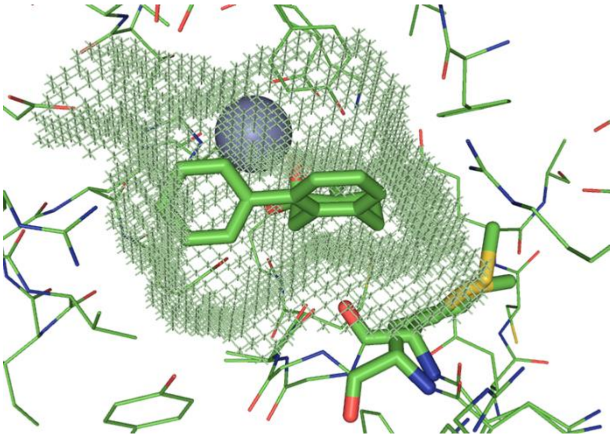

(a)

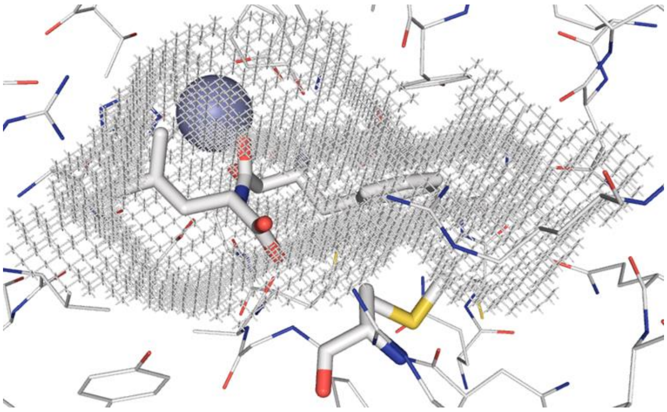

(b)

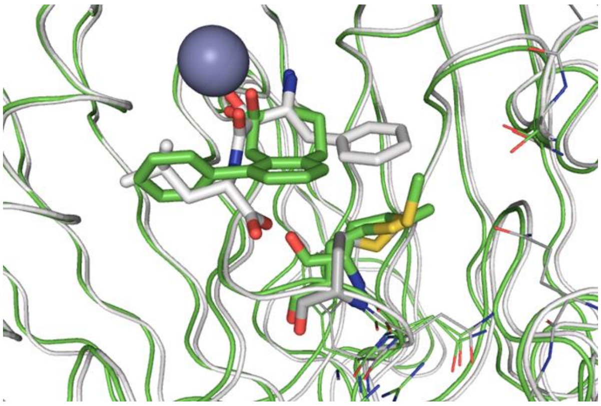

(c)

Figure S4. Local movement of Met260 in the active site of *Ec*PepN. (a) Surface representation of the active site of *Ec*PepN in complex with (a) aminobenzosuberone **21c** (green, PDB 5MFS) and (b) bestatin **1** (white, PDB 2DQM). (c) Superimposition of the protein backbone of *Ec*PepN in complex with aminobenzosuberone **21c** (green, PDB 5MFS) and bestatin **1** (white, PDB 2DQM). The active site

cavity was computed by KVFinder. The active site residues are represented in line and the zinc ion is shown as grey sphere. The key Met260 residue involved in S1 subsite modulation was represented in stick. Images generated with PyMOL.

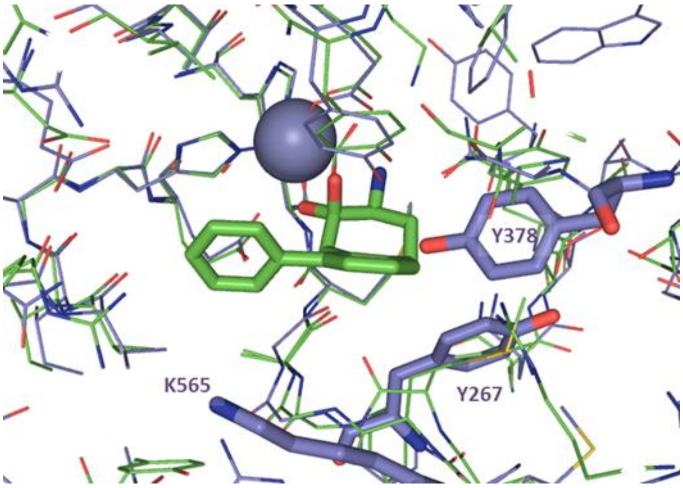

Figure S5. Superimposition of the protein backbone of the aminobenzosuberone **21c** in *Ec*PepN complex (green, PDB 5MFS) with *Hs*LTA$_4$H (slate blue, PDB 1HQ6). The shorter distance between the oxygen atom of *Hs*LTA$_4$H Tyr378 and the aromatic core of aminobenzosuberone **21c** was 1.2Å. This close proximity may induce severe steric clashes causing the large loss of potency observed for *Hs*LTA$_4$H inhibition. The active site residues are represented in line and the zinc ion is shown as grey sphere. The key residues involved in *Hs*LTA$_4$H's S1 subsite modulation were represented in stick. Images generated with PyMOL.

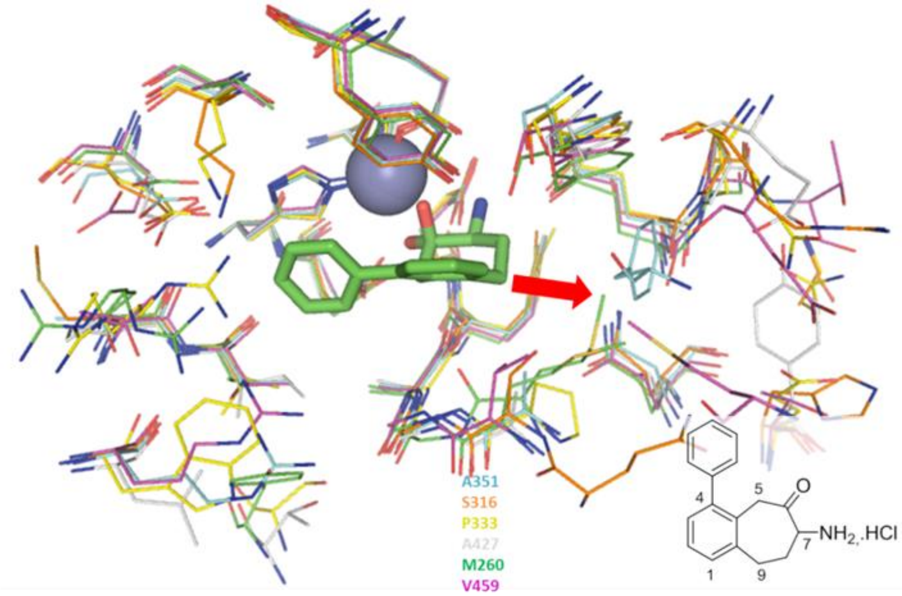

Figure S6. Superimposition of the protein backbone of the aminobenzosuberone **21c** in *Ec*PepN complex (green, PDB 5MFS) with different M1 aminopeptidases. *Hs*APN (cyan, PDB 4FYT), *Hs*ERAP1 (orange, PDB 2YD0), *Hs*ERAP2 (yellow, PDB 4JBS), *Hs*IRAP (white, PDB 5MJ6), *Pf*AM1 (magenta, PDB 3EBH). The catalytic zinc ion is shown as grey sphere. The gatekeeper residue is listed. Images generated with PyMOL.

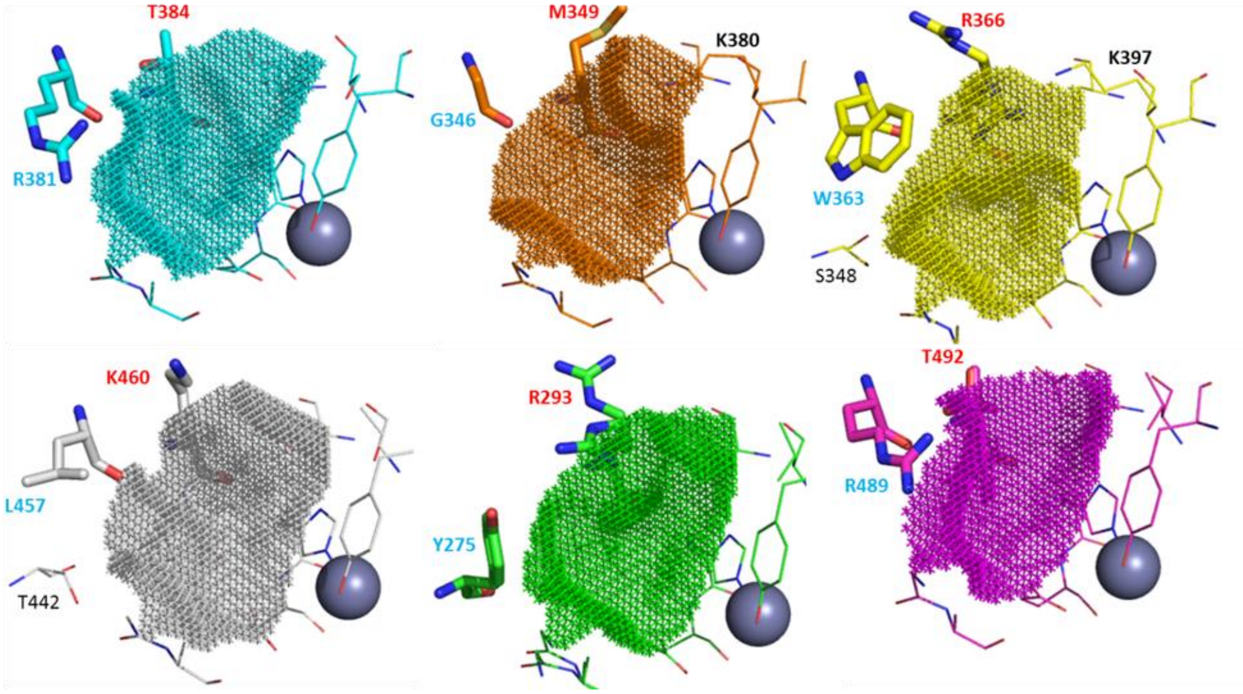

Figure S7. Surface representation of the S1′ subsite of different M1 aminopeptidases. *Hs*APN (cyan, PDB 4FYT), *Hs*ERAP1 (orange, PDB 2YD0), *Hs*ERAP2 (yellow, PDB 4JBS), *Hs*IRAP (white, PDB 5MJ6), *Ec*PepN (green, PDB 5MFS), *Pf*AM1 (magenta, PDB 3EBH). The catalytic zinc ion is shown as grey sphere. Residues involved in S1′ plasticity are shown in stick. In blue, residues which reduce the cavity width; in red, residues which modulate the cavity depth. Images generated with KVFinder plugin in PyMOL.

Table S4. Cavity volume of the S1′ subsite of "closed" conformation of M1 APs

<table>
<tr><th colspan="2">« closed » conformation of M1 AP</th><th>ligand</th><th>PDB entry</th><th>Volume (Å^3)</th></tr>
<tr><td rowspan="2">HsAPN</td><td rowspan="2">local movement in S1′ subsite: motion of R381 residue</td><td>AMA</td><td>4FYT</td><td>291.97</td></tr>
<tr><td>1</td><td>4FYR</td><td>271.81</td></tr>
<tr><td>HsERAP1</td><td></td><td>1</td><td>2YD0</td><td>330.30</td></tr>
<tr><td rowspan="2">HsERAP2</td><td rowspan="2">local movement in S1′ subsite:
motion of R366</td><td>APA</td><td>5AB0</td><td>294.40</td></tr>
<tr><td>PPT1</td><td>4JBS</td><td>247.62</td></tr>
<tr><td>HsIRAP</td><td></td><td>PPT2</td><td>5MJ6</td><td>369.10</td></tr>
<tr><td rowspan="2">EcPepN</td><td rowspan="2">local movement in S1′ subsite:
motion of R293 residue</td><td>21c</td><td>5MFS</td><td>291.65</td></tr>
<tr><td>1</td><td>2DQM</td><td>276.03</td></tr>
<tr><td>PfAM1</td><td></td><td>1</td><td>3EBH</td><td>301.44</td></tr>
</table>

The cavity volume was calculated using KVFinder plugin in PyMOL. Ligand abbreviations stand for : AMA amastatin, APA aminophosphonic acid, PPT1/2 phosphinic pseudotripeptides. In blue, residues which reduce the cavity width; in red, residues which modulate the cavity depth.

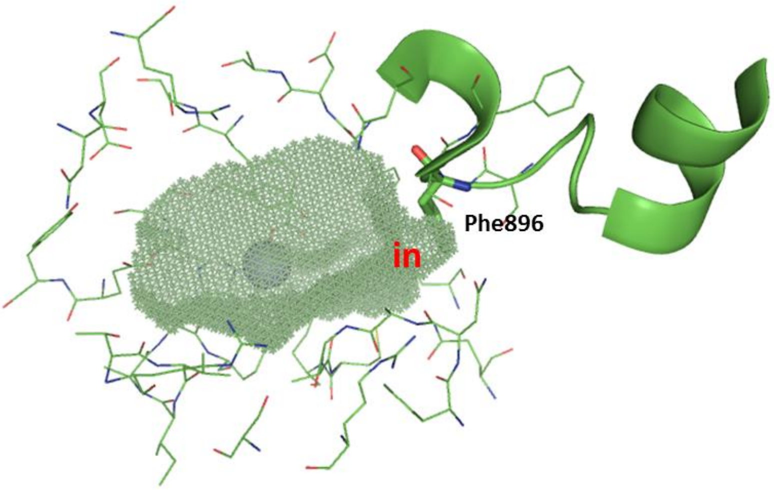

(a)

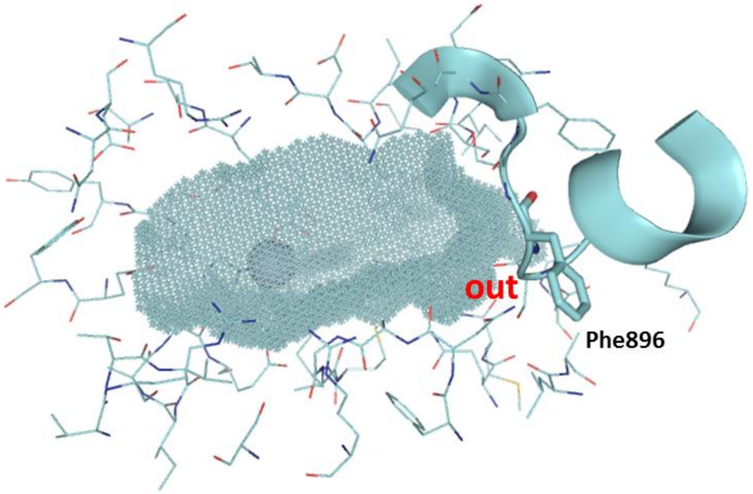

(b)

Figure S8. Intra-domain movement in the active site of *Hs*APN . Surface representation of the active site in both determined *Hs*APN structures for (a) PDB 4FYT (green, cavity volume 572.12 Å^3) and (b) PDB 4FYR (cyan, cavity volume 811.87 Å^3). In the "Phe-In" conformation, Phe896 is oriented into the active site inducing a partial closing of the S1 subsite. The active site cavity was computed by KVFinder. The flexible 891-998 loop was represented in cartoon and the key Phe896 residue involved in S1 subsite modulation was represented in stick.

Table S5. Determination and prediction of various ADME-Tox properties of substituted 7-amino-5,7,8,9-tetrahydrobenzocyclohepten-6-one hydrochloride salts.

Compound	$logD_{7.4}$	BBB	P-gp	Inhibition CYP450				hERG	H-HT	AMES
				1A2	3A4	2C9	2D6			
**21a**	0.54	0.993	0.5	0.311	0.041	0.05	0.334	0.147	0.304	0.238
**21b**	1.28	0.977		0.206	0.066	0.051	0.327	0.17	0.282	0.204
**21c**	2.12	0.982		0.567	0.149	0.223	0.398	0.706	0.444	0.366
**21d**	-	-		-	-	-	-	-	-	-
**21e**	1.41	0.977		0.311	0.032	0.061	0.375	0.15	0.154	0.204
**21f**	2.22	0.982		0.656	0.097	0.172	0.411	0.709	0.448	0.366
**21g**	1.67	0.987		0.707	0.058	0.076	0.405	0.28	0.518	0.382
**21h**	2.48	0.977		0.15	0.027	0.072	0.326	0.162	0.162	0.204

**21i**	3.12	0.967		0.517	0.144	0.27	0.383	0.78	0.484	0.302
**21j**	3.06	0.967		0.452	0.138	0.361	0.406	0.784	0.548	0.302

$logD_{7.4}$ is the distribution coefficient experimentally determined at pH 7.4; the TPSA (Topological Polar Surface Area) value for the whole series of aminobenzosuberone is 44.71 $Å^2$ and 68.10 $Å^2$ for the ketone and hydrate form, respectively; for the following ADME-Tox properties, liability is set above a threshold value of 0.1 for BBB permeation, and above 0.5 for other characteristics. Value closer to 1 means high liability risk. BBB is the probability of blood-brain barrier permeation; P-gp is the probability of efflux by P-gp transporter pump; 1A2, 3A4, 2C9, 2D6 are the probability of inhibition of the different CYP450 isozymes; hERG is the probability of blocking the hERG channel; H-HT is the probability of human hepatotoxicity; AMES is the probability of Ames mutagenicity.
